# Aerial strategies advance volcanic gas measurements at inaccessible, strongly degassing volcanoes

**DOI:** 10.1126/sciadv.abb9103

**Published:** 2020-10-30

**Authors:** E. J. Liu, A. Aiuppa, A. Alan, S. Arellano, M. Bitetto, N. Bobrowski, S. Carn, R. Clarke, E. Corrales, J. M. de Moor, J. A. Diaz, M. Edmonds, T. P. Fischer, J. Freer, G. M. Fricke, B. Galle, G. Gerdes, G. Giudice, A. Gutmann, C. Hayer, I. Itikarai, J. Jones, E. Mason, B. T. McCormick Kilbride, K. Mulina, S. Nowicki, K. Rahilly, T. Richardson, J. Rüdiger, C. I. Schipper, I. M. Watson, K. Wood

**Affiliations:** 1 University College London, London WC1E6BS, UK.; 2 University of Cambridge, Cambridge CB23EQ, UK.; 3 Università di Palermo, 90123 Palermo, Italy.; 4 GasLAB, Universidad de Costa Rica, San José, Costa Rica.; 5 Chalmers University of Technology, Göteborg, Sweden.; 6 Heidelberg University, Heidelberg, Germany.; 7 Max Planck Institute for Chemistry, Mainz, Germany.; 8 Michigan Technological University, Houghton, MI 49931, USA.; 9 University of Bristol, Bristol, BS8 1TR, UK.; 10 Universidad Nacional, Heredia, 40101-3000 Costa Rica.; 11 University of New Mexico, Albuquerque, NM 87131, USA.; 12 University of Saskatchewan, Centre for Hydrology, Canmore, Alberta T1W 3G1, Canada.; 13 INGV, Osservatorio Etneo, Sezione di Catania, 95125 Catania, Italy.; 14 Johannes Gutenberg-Universität, Mainz 55128, Germany.; 15 University of Manchester, Manchester, M13 9PL, UK.; 16 Rabaul Volcanological Observatory, Rabaul, Papua New Guinea.; 17 Victoria University of Wellington, Wellington 6012, New Zealand.

## Abstract

Volcanic emissions are a critical pathway in Earth’s carbon cycle. Here, we show that aerial measurements of volcanic gases using unoccupied aerial systems (UAS) transform our ability to measure and monitor plumes remotely and to constrain global volatile fluxes from volcanoes. Combining multi-scale measurements from ground-based remote sensing, long-range aerial sampling, and satellites, we present comprehensive gas fluxes—3760 ± [600, 310] tons day^−1^ CO_2_ and 5150 ± [730, 340] tons day^−1^ SO_2_—for a strong yet previously uncharacterized volcanic emitter: Manam, Papua New Guinea. The CO_2_/S_T_ ratio of 1.07 ± 0.06 suggests a modest slab sediment contribution to the sub-arc mantle. We find that aerial strategies reduce uncertainties associated with ground-based remote sensing of SO_2_ flux and enable near–real-time measurements of plume chemistry and carbon isotope composition. Our data emphasize the need to account for time averaging of temporal variability in volcanic gas emissions in global flux estimates.

## Teaser

Aerial measurements using unoccupied aerial systems (UAS) transform our ability to measure and monitor volcanic plumes.

## INTRODUCTION

Volcanoes are an important pathway for the transfer of volatiles from Earth’s interior into the atmosphere and oceans, representing an intersection between Earth’s deep and shallow carbon cycles (*1*–*3*). The chemical and isotopic compositions of volcanic emissions provide critical insights into the source(s) of emitted volatiles (i.e., mantle-, crust-, or slab-derived) (*4*–*7*), as well as real-time indications of the conditions of magma storage and degassing (i.e., pressure, temperature, and oxidation state) (*8*–*10*). Measurements of volcanic gases at the surface are therefore critical to both volcano monitoring and to the robust quantification of global volatile budgets, and yet, volcanic CO_2_ fluxes into the atmosphere remain highly uncertain. High-background CO_2_ concentrations present challenges for sensitive detection by remote sensing, and the need to collect undiluted gas samples to analyze carbon isotopes necessitates proximal plume access. These sampling limitations have biased estimates of global carbon flux and carbon sources toward a relatively small number of accessible, passively degassing volcanoes (*7*, *11*–*13*). At present, constraints on carbon degassing exist for ~60 of the ~300 currently active volcanoes and, of those, only ~10 are characterized by long-term datasets that enable any assessment of temporal variability in gas composition or carbon emission rates (*11*). SO_2_ emissions, in contrast, can be readily detected and quantified by satellite (*14*–*16*) and ground-based (*16*–*20*) remote sensing. By enabling proximal sampling of remote or hazardously accessible volcanic plumes, instrumented unoccupied aerial systems (UAS) are now targeting gaps in our knowledge of carbon degassing at some of the major remaining “known unknown” volcanic emitters.

Aerial robotic strategies using UAS are changing the landscape of volcanological research and monitoring, contributing accurate and repeatable data at spatial resolutions often exceeding ground- or space-based equivalents (*21*). Proximal gas measurements with instrumented UAS build on the advances made by conventional crewed aircraft surveys at several remote volcanoes (*22*–*27*), arguably contributing a more accessible, flexible, cost-effective, and lower-risk strategy for these environments. For example, although small UAS (i.e., <200-kg total takeoff weight) can vary considerably in cost—from less than £2000 for a small multirotor, to ~£5000 for a custom (hobbyist) fixed-wing build, to more than £15,000 for some commercial builds—when compared to the costs of crewed aircraft flight time (which can have operating costs of thousands of pounds per flying hour plus >£100,000 in facility setup; e.g., Facility for Airborne Atmospheric Measurements aircraft, UK MET Office personal communication), the cost saving is substantial and promotes repeat time series measurements, although the range and payload capability between the two vehicles are not strictly comparable. While initial applications of UAS centered largely on remote imaging and the derivatives thereof, further advances in UAS technology (combined with ever-increasing affordability) together with concurrent efforts to miniaturize instruments have facilitated community-wide progress toward a more comprehensive suite of in situ measurements and sampling, which includes gas sensing applications (*28*–*33*). Short-range aerial gas sensing has now become sufficiently mature to enable integration within either regular volcano monitoring procedures or crisis response situations (*34*–*39*). However, beyond-visual-line-of-sight (BVLOS) operations, where the UAS is operating out of the view of either the pilot or an intermediate observer, have rarely been attempted in volcanic environments (*38*, *40*). Further, there have been few attempts to acquire simultaneously ground-based and aerial gas measurements (*41*).

**Fig. 1:**
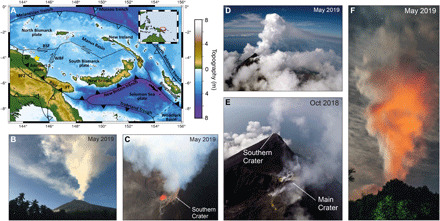
Aerial Observations of Manam, Papua New Guinea. (**A**) Regional tectonic setting. Manam is located within the West Bismarck Volcanic Arc (yellow star). (**B**) The more energetic, high-altitude plume from the Southern Crater often dispersed in a different direction to the weaker, low-altitude emissions from the Main Crater. Image taken on 25 May 2019. (**C**) A nadir image acquired during a UAS overpass on 22 May 2019 showed that magma was present at shallow levels within the Southern Crater. A strong plume emanated from the crater. (**D**) View from UAS during plume approach. The buoyant plume from the Southern Crater rose to ~2 to 3 km above sea level before dispersing laterally. (**E**) Aerial view of the summit showing persistent passive degassing from the Southern Crater (behind the summit in this view) and the broader Main Crater area, acquired during a UAS flight on 30 October 2018 at 21:00 UTC (07:00 local time). (**F**) Strong nighttime incandescence reflected by the rising plume above the Southern Crater on 25 May 2019, viewed from Baliau village. Image credits: (B) E. J. Liu; (C to E) K. Wood, pilot; and (F) M. Wordell.

Manam (4.080°S, 145.037°E) is a basaltic stratovolcano in the Western Bismarck volcanic arc, located ~13 km off the northeast coast of mainland Papua New Guinea (Fig. 1A). The subaerial edifice rises 1800 m above sea level (asl) forming a near-circular island ~10 km in diameter. Manam erupts mafic rocks that are petrologically similar to tholeiitic basalts yet characterized by extremely low TiO_2_ contents (*42*). The tectonic setting of the region is complex, dominated by oblique northeast-southwest plate convergence (Fig. 1A). Manam is located in the segment of the Bismarck volcanic arc where arc-continent collision took place in the late Miocene to Pliocene during closure of the Solomon Sea (*43*–*46*). This suturing of arc and continent destroyed the submarine trench and is consistent with a hanging slab that has been detected by seismic tomography at about 100-km depth below the north coast ranges (*47*, *48*). Manam is one of the most active volcanoes in Papua New Guinea since historical records began (*49*, *50*), characterized by persistent passive degassing and intermittent Strombolian activity, punctuated by paroxysmal sub-Plinian eruptions on subdecadal time scales. A major eruption beginning in October 2004 culminated in a climactic explosive event on 27 January 2005 that injected ash to stratospheric heights of 21 to 24 km (*51*). Together with an eruption in 2006, again with emissions into the stratosphere, these events devastated large sectors of the island and displaced the island population to the mainland (*52*). Mild to moderate explosive activity has continued sporadically at Manam since the 2004 to 2006 eruptions, with the current phase of eruptive activity beginning in June 2014 (*53*).

Manam is currently ranked among the strongest volcanic emission sources globally. Satellite measurements of SO_2_ emissions from Manam between 2005 and 2015 indicate an average SO_2_ flux (*16*) of 1480 ± 750 [1σ] tons day^−1^. However, despite a historical record of persistent passive degassing, frequent explosive activity, and globally significant SO_2_ emissions, there exists no prior constraint on carbon degassing at Manam from in situ measurements. Global relationships between the composition of volcanic gases and petrological proxies (e.g., whole-rock trace element compositions; Ba/La) predict the mean CO_2_/S_T_ at Manam to be 2.7 ± 0.7 (*11*). Combined with satellite-based estimates of long-term SO_2_ flux (*15*), Aiuppa *et al.* (*11*) predicted the emission rate of CO_2_ to be 2760 ± 1570 tons day^−1^ or ~1 Mt CO_2_/year during 2005 to 2015, placing Manam among the most significant volcanic carbon sources currently active.

Here, we integrate multiscale measurements from ground-based remote-sensing, aerial measurements using instrumented UAS and satellite observations to derive the first multispecies gas fluxes for Manam volcano. We expand the known capabilities of UAS to include in situ measurements of gas composition, spectroscopic and wind speed measurements to derive SO_2_ flux, and retrievable bag samples of plume gases for carbon isotope measurements. We use these techniques in tandem during two field campaigns at Manam (30 to 31 October 2018 and 20 to 27 May 2019) to characterize the emissions from this strongly degassing volcano. By combining measured molar gas ratios with independent constraints on SO_2_ flux, we test the predicted carbon flux based on trace element relationships (*11*). Our novel approach—that is, long-range and high-altitude UAS operations enabling in situ measurements—is presently the only feasible means by which we can characterize gas chemistry at steep, hazardous, and highly active volcanoes like Manam. Our success in both measuring and sampling volcanic gases using UAS demonstrates the potential of aerial strategies to transform our ability to monitor emissions from active volcanoes globally.

## RESULTS

### Recent activity and direct observations

**Fig. 2:**
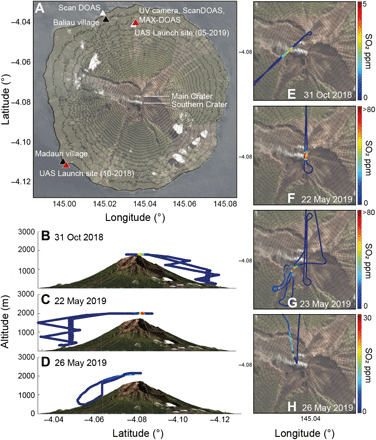
Instrument locations and flight paths. (**A**) The positions of the Main and Southern Craters are annotated and correspond to the two white plumes visible on the satellite image. The four avalanche valleys that dominate the local topography radiate from the summit area. Launch and landing sites for UAS flights in October 2018 and May 2019 are indicated by the red triangles located in the southwest and north of the island, respectively. The positions of static ground-based instruments are indicated by the annotated white triangles. Black triangles show the location of the village communities nearest to each of the measurement locations. Elevation contours at 50-m intervals (extracted from WorldDEM, Airbus Space and Defence) are superimposed on a satellite image of Manam Island (courtesy of Planet Labs Inc.). (**B** to **D**) Lateral view of selected UAS flight tracks, showing a vertical ascent of >2000 m. (**E** to **H**) Top-down view of UAS flight tracks colored according to georeferenced SO_2_ concentrations; warmer colors correspond to higher SO_2_ concentrations up to ~100 ppm. The UAS intersected a vertically ascending plume directly over the Southern Crater on 22 May 2019. In contrast, the plume was more strongly influenced by a north-easterly wind on 23 May 2019, requiring a change to manual (rather than automated waypoint) piloting at plume altitude to ensure plume intersection. In May 2019, the altitude of fixed-wing UAS overpasses (2300 m asl) was too high to intersect the weaker emissions that emanated from the northerly Main Crater at an altitude of ~1800 m asl.

Manam has two active vents—Main Crater and Southern Crater—situated on a broad summit plateau elongated in the north-south direction (Figs. 1 and 2). The two vents have been active simultaneously throughout much of the last century, although most of the explosive activity since 1945 has been focused at the Southern Crater (*49*). In Sentinel-2 satellite imagery, one or two thermal anomalies per clear-sky observation have been detected repeatedly since observations began in 2016 (fig. S1), corresponding to the positions of the two summit vents. Here, a thermal anomaly is identified on the basis of the difference in spectral intensity between two wavelength bands—2202.4 nm (band 12, shortwave infrared) and 864.7 nm (band 8A, near infrared)—that are usually correlated except in the presence of a thermal emission source. A major eruption on 25 August 2018 from the Southern Crater generated a 15-km-high eruption column and initiated lava flows from the Main Crater into the northeast avalanche valley, which continued until 12 October 2018 (fig. S1) (*53*). This eruption signaled the start of a new phase of elevated activity after a period of relative quiescence since the previous Strombolian eruptions in early 2017. Further moderate to large explosive eruptions occurred on 30 September 2018, 8 December 2018, 8 and 24 January 2019, 28 June 2019, and, most recently, 6 November 2019. Intereruptive periods were characterized by persistent, strong, passive degassing (*53*). Following the two closely spaced eruptions in January 2019, the thermal anomaly at the Main Crater disappeared from subsequent satellite imagery (fig. S1). Correspondingly, degassing from the Main Crater was noticeably reduced during a field campaign in May 2019, compared to that observed in October 2018. After 9 months of absence, a thermal anomaly was again detected at the Main Crater on 17 October 2019, only 2 weeks before a 9.5-hour-long eruption involving a sustained lava fountain at the Main Crater (fig. S2).

Direct aerial observations of the summit were made during UAS overpasses on 30 October 2018 and 20 to 27 May 2019 (Figs. 1 and 2). Observations in October 2018 directly followed a cessation of a prolonged period of explosive and effusive activity, while those in May 2019 preceded a major eruption 1 month later on 28 June 2019 (fig. S1). In October, freshly emplaced lava flows were observed originating from the northeast margin of the Main Crater. Two distinct plumes were visible, corresponding to emissions from both the Main and Southern Craters. Degassing sources at the Main Crater were broadly distributed in the form of numerous small, sulfur-encrusted vents. However, the deep regions of the craters were obscured by condensed gas emissions. By May 2019, degassing had focused at the Southern Crater and intensified. A nadir image of the Southern Crater taken on 22 May 2019 (Fig. 1C) during non-condensing plume conditions showed that the top of the magma column was at most a few hundred meters below the ground surface. The presence of magma at a shallow level was supported by strong nighttime incandescence, such as observed on 25 May 2019 (Fig. 1F) and by the presence of a single strong thermal anomaly in Sentinel-2 imagery acquired on 20 May 2019 (fig. S1). An energetic, thermally buoyant gas plume emanated from the magma surface and generally rose to heights of 1 to 3 km above the summit before dispersing laterally. Weaker, but still persistent, degassing was observed from numerous small, sulfur-encrusted vents in the Main Crater region, near the upper part of a collapse scar on the eastern flank. These fumaroles fed a less energetic, low-altitude plume that generally migrated laterally at summit altitude. The high- and low-altitude plumes were often observed moving in different directions (Fig. 1B), indicating a heterogeneous vertical wind profile above the volcano.

### Aerial measurements of gas composition

In situ measurements of plume composition were acquired using aerial multi-component gas analysis systems (Multi-GAS; see Materials and Methods) mounted on both fixed-wing and multirotor UAS (fig. S3). Full details of sensor specifications and data processing are provided in Materials and Methods. A long-range fixed-wing flight to 2000 m asl (200 m above summit altitude) sampled dilute emissions from the Main Crater on 30 October 2018 (Fig. 2D). Subsequent flights using the same vehicle and sensor payload on 22 and 23 May 2019 intercepted the ascending region of the strong, higher-altitude plume from the Southern Crater at a height of 2300 m asl (500 m above summit altitude). Gas concentrations are greatest in the central region of the plume; direct interceptions of the dense, rising plume yields higher concentration measurements with enhanced signal-to-noise—and thus reduced uncertainties on derived gas ratios—compared to similar measurements from a dilute downwind plume. Each traverse through the dense region of the vertically rising plume lasted approximately 25 ± 4 s (based on the average ± σ of five traverses), using SO_2_ as the plume marker. At a ground speed of 26.6 ± 1.4 [σ] m s^−1^, this travel time corresponds to an average plume diameter of 665 ± 112 m, during which the UAS experienced extreme turbulence. Weaker emissions from the Main Crater were dispersing at an altitude too low to be safely intercepted in May 2019. A multirotor UAS flight on 26 May 2019 intercepted the vertically rising plume from the Southern Crater at 2300 m asl.

**Fig. 3:**
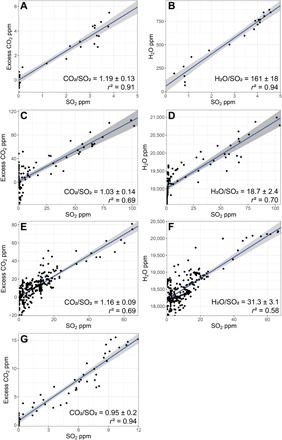
Volcanic molar gas composition. CO_2_-SO_2_ and H_2_O-SO_2_ regression scatterplots for (**A** and **B**) 30 October 2018 21:00 UTC (07:00 local time), (**C** and **D**) flight 1 on 22 May 2019 06:30 UTC (16:30 local time), (**E** and **F**) flight 2 on 23 May 2019 00:00 UTC (10:00 local time), and (**G**) flight 4 on 26 May 2019 00:00 UTC (10:00 local time). CO_2_ is shown as “excess,” where the background is taken as the *y*-axis intercept of the regression line. There is high variability in CO_2_ at low SO_2_ (dilute plume) conditions. Molar gas ratios are determined by least squares linear regression (solid blue line). Goodness of fit is shown by the adjusted *r*^2^ values. Gray shaded region represents the 95% confidence bounds on the regression. Data are from two Multi-GAS instruments: (A to F) Università di Palermo and (G) Chalmers University (see Materials and Methods for specifications).

The molar CO_2_/SO_2_ and H_2_O/SO_2_ ratios of Main Crater emissions measured in October 2018 were 1.19 ± 0.13 and 161 ± 18, respectively (Table 1; error represents 95% confidence intervals on the regression), at the H_2_O-rich end of typical high-temperature magmatic emissions at arc volcanoes (*12*, *54*). These ratios translate to molar proportions of 98.7 mol % H_2_O, 0.7 mol % CO_2_, and 0.6 mol % SO_2_. The CO_2_/SO_2_ ratios of Southern Crater emissions measured in May 2019 ranged between 0.95 and 1.16, with a mean of 1.07 ± 0.06 from four measurements (Fig. 3 and Table 1). H_2_O/SO_2_ ratios were 18.7 ± 2.4 and 31.3 ± 3.1 from two measurements. Corresponding molar gas compositions were 90.2–93.5 mol % H_2_O, 3.5–5 mol % CO_2_, and 3–4.8 mol % SO_2_. Sulfur as hydrogen sulfide (H_2_S), typically the dominant sulfur species in reducing, low-temperature emissions [often associated with hydrothermal systems (*55*, *56*)], was not measured above the detection limit (see Materials and Methods) in any of the acquisitions. For comparison to other datasets, X/SO_2_ ratios are therefore equivalent to X/S_T_, where X refers to the species of interest (for example, CO_2_ or H_2_O) and S_T_ refers to total sulfur (SO_2_ + H_2_S).

**Table 1:** Volcanic gas compositions, expressed as molar ratios and molar proportions. Reported uncertainties on molar ratios are 95% confidence bounds (1.96 × standard error of the regression). Uncertainties on flux measurements are asymmetrical and therefore quoted as ± [upper/lower bounds]. H_2_S was not detected above the 13% cross-sensitivity of the sensor to SO_2_. The SO_2_ flux reported for each compositional measurement is the average of all flux measurements made on the same day by multiple techniques (see Table 2).

**Date/Time (UTC)**		**30 October** **2018/21:00**	**22 May** **2019/06:30**	**23 May** **2019/00:00**	**23 May** **2019/00:45**	**26 May** 2019*/00:00	**Average (May 2019) ±** **propagated error**
**Vent sampled**		Main Crater	Southern Crater	Southern Crater	Southern Crater	Southern Crater	
**Molar**	CO_2_/SO_2_ (±2σ)	1.19 ± 0.13	1.03 ± 0.14	1.16 ± 0.09	1.12 ± 0.12	0.95 ± 0.10	1.07 ± 0.06
	*r*^2^	0.91	0.69	0.69	0.96	0.94	
	H_2_O/SO_2_ (±2σ)	161 ± 18	18.7 ± 2.4	31.3 ± 3.1	–	–	
	*r*^2^	0.94	0.7	0.58			
	BrO/SO_2_ (×10^−5^ ± 2σ)	–	1.80 ± 0.03	1.20 ± 0.08	1.20 ± 0.08	1.20 ± 0.03	2.02 ± 0.04
	*r*^2^	–	0.87	0.74	0.74	0.8	
**Mass**	CO_2_/SO_2_	0.82 ± 0.09	0.71 ± 0.10	0.80 ± 0.06	0.77 ± 0.08	0.65 ± 0.07	
	H_2_O/SO_2_	45.3 ± 5.1	5.3 ± 0.7	8.8 ± 0.9	–	–	
	BrO/SO_2_ (×10^−5^)		2.69 ± 0.04	1.80 ± 0.6	1.80 ± 0.6	1.80 ± 0.04	
**Molar** **composition**	H_2_O (mol%)	98.7	90.2	93.5	–	–	
	CO_2_ (mol%)	0.7	5	3.5	–	–	
	SO_2_ (mol%)	0.6	4.8	3	–	–	
	BrO (×10^−7^ mol%)	–	8.7	3.6	–	–	
**Mass flux**	SO_2_ flux (tons day^−1^)	–	5825 ± [927/987]	4900 ± [346/1816]	4900 ± [346/1816]	4973 ± [841/1015]	5150 ± [336/733]
	CO_2_ flux (tons day^−1^)	–	4122 ± [863/896]	3905 ± [410/1479]	3770 ± [484/1455]	3245 ± [646/745]	3760 ± [313/595]
	H_2_O flux (×10^3^ tons day^−1^)	–	30.6 ± [6.3/6.5]	43.1 ± [5.2/16.2]			36.9 ± [4.1/8.9]
	BrO flux (×10^−2^ tons day^−1^)	–	16 ± [0.3/0.3]	8.8 ± [0.9/3.3]	8.8 ± [0.9/3.3]	8.9 ± [1.5/1.8]	10 ± [0.8/1.4]

*This measurement was made using a different multi-gas instrument from the preceding dates. See Materials and Methods for full details.

Comparing measured gas compositions in October 2018 (Main Crater) and May 2019 (Southern Crater), we find that the CO_2_/S_T_ ratios were similar within uncertainty and both within the modal range of CO_2_/S_T_ values from high-temperature (≥ 450°C) arc emissions globally (*6*). In contrast, H_2_O/S_T_ differed by an order of magnitude between the two campaigns. From the available data, we cannot resolve unambiguously whether this compositional change represents a real temporal shift in emitted gas composition or if it instead reflects spatial heterogeneity between the two summit vents. However, visual observations indicate that the Main Crater is dominantly fumarolic in comparison to the open-vent situation of the Southern Crater, despite the lack of detected H_2_S. Intuitively, sulfur scrubbing by a shallow hydrothermal system at the Main Crater would explain the difference in H_2_O/S_T_ but is not consistent with the similar CO_2_/S_T_ ratios. Alternatively, a contribution from meteoric water in fumarolic emissions could explain the elevated water contents (>98 mol%) in the October measurements with no change in CO_2_/S_T_, but hydrogen and oxygen isotope measurements would be needed to confirm this definitively.

BrO/SO_2_ ratios, measured remotely using ground-based Multi-axis Differential Optical Absorption Spectroscopy (MAX-DOAS; full details are given in Materials and Methods), range from 6.6 × 10^−6^ to 4.7 × 10^−5^ across a measurement period of 6 days, with a median value of 1.5 × 10^−5^ ± 6.2 × 10^−6^ (standard error of the median; fig. S4 and table S1). The correlation between BrO and SO_2_ is poor for 2 of the 6 days, and we attribute this to the presence of two different emission sources (and thus a variably mixed plume) that could not be differentiated from the ground-based viewing angle of the instrument. However, we cannot exclude the possibility that some of the decorrelation between these two species is derived from the need to use a fixed solar reference spectrum (see Materials and Methods).

### Aerial and ground-based constraints on SO_2_ flux

**Fig. 4:**
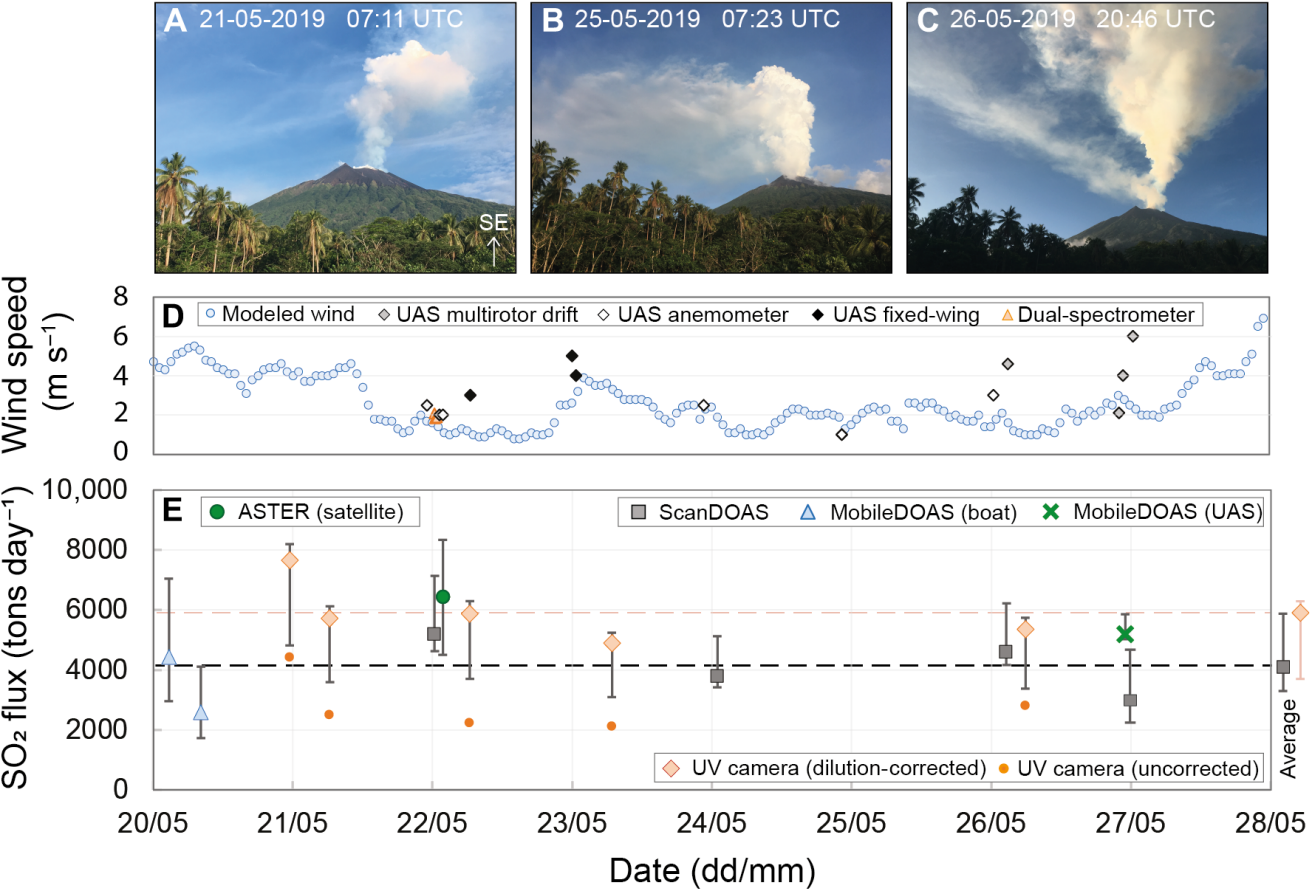
SO_2_ flux measurements. (**A** to **C**) Variability in plume height and direction during 21 to 27 May 2019. All images were taken from the location of the fixed scanning differential optical absorption spectrometry (ScanDOAS) instrument in Baliau (Fig. 2A), looking southeast. Image credit: E. J. Liu. (**D**) Summary of wind speeds in the horizontally dispersing plume measured directly by various techniques (see Materials and Methods) or modeled, assuming plume transport at 2000 m above summit altitude. (**E**) Summary of SO_2_ flux measurements at Manam during 20 to 27 May 2019. For the UV camera and ScanDOAS data, multiple measurements were acquired in a single sampling interval, and therefore, each point represents the mean value ± measurement uncertainty (see Materials and Methods). Dashed lines indicate the mean values over the whole campaign for UV camera (orange dashed line) and all DOAS combined (black dashed line); propagated uncertainties on the average values are shown on the right-hand side of the figure.

A summary of SO_2_ emission rates measured between 20 and 27 May 2019 is presented in Fig. 4 and Table 2. Ultraviolet (UV) camera measurements, acquired from the Godagi cone in the north of the island (200 m asl; Fig. 2) at a distance of ~5 km from the vent, indicate SO_2_ fluxes ranging from 4900 ± [350, 1820] tons day^−1^ to 7660 ± [540, 2840] tons day^−1^, with a mean of 5900 ± [420, 2220] tons day^−1^ (where asymmetric errors represent the uncertainty on the measurements and are represented as ± [upper/lower]). Considering only fully clear image sequences (the plume was partially obscured by cloud cover on 23 and 26 May 2019), the mean flux is slightly higher at 6420 ± [460, 2400] tons day^−1^. UV camera data are explicitly corrected for light dilution using the method presented by Campion *et al.* (*57*), which increases the derived flux by 74 to 160%. The large negative errors for UV camera SO_2_ fluxes reflect the difference between raw and corrected fluxes (both shown in Fig. 4). Calculated plume speeds, used to derive emission rates from SO_2_ integrated column amounts (ICAs), range from 5 ± 1 to 17 ± 5 m s^−1^ in the vertically ascending plume immediately above the vent [speeds estimated using optical flow, a feature tracking algorithm (*58*); see Materials and Methods]. As a result of the often-diverging plumes from the Southern and Main Craters (e.g., Fig. 1B), some regions of the plume were obscured to varying degrees by either topography or cloud cover on all days apart from 20 May 2019, where both plumes were captured fully within the field of view.

**Table 2:** Summary of SO_2_ flux measurements. Uncertainties on flux measurements are asymmetrical and therefore quoted as ± [upper/lower bounds].

**Date (UTC)**	**Mean time (UTC)**	**Measurement** **duration, min** **(number of scans)**	**Mean plume speed** **(m s^−1^)**	**Mean SO_2_ flux** **(tons day^−1^)**	**± SD (1σ)**	**Notes on plume** **condition**
**UV camera**						
20 May 2019	23:30	120	9.0 ± 1.9	7660 ± 541/2838	1930	Very clear, entire plume captured
21 May 2019	06:20	160	5.0 ± 0.8	5710 ± 404/2118	1100	Very clear, plume partially obscured by flank
22 May 2019	06:22	44	16.6 ± 4.6	5880 ± 416/2181	1900	Plume slightly covered by cloud
23 May 2019	06:52	34	16.8 ± 5.1	4900 ± 346/1816	1100	Plume partially covered by cloud
26 May 2019	05:52	65	10.9 ± 5.6	5360 ± 379/1986	3180	Plume partially covered by cloud
Average				5900 ± 423/2215		
Average (clear plume only)				6420 ± 458/2400		
**ScanDOAS**						
22 May 2019	00:23	(43)	1.5 ± 0.2	5180 ± 1966/564	3900	Only plumes with complete coverage and close proximity to scanner azimuth were selected to minimize uncertainty in wind direction
24 May 2019	00:57	(72)	1.8 ± 0.5	3770 ± 1362/360	1461	
26 May 2019	02:36	(84)	1.4 ± 0.3	4590 ± 1638/423	1340	
27 May 2019	00:58	(30)		3033 ± 2038/1024	1190	
**Boat traverse (MobileDOAS)**						
20 May 2019	02:43	(30)	3	4440 ± 2607/1479	NA	
20 May 2019	08:14	(30)	3	2590 ± 1521/868	NA	
**UAS traverse (MobileDOAS)**						
26 May 2019	23:00	(10)	6	5200 ± 657/179	NA	
Average (all DOAS)				4115 ± 1777/814		

SO_2_ emission rates were also determined independently by DOAS. ICAs of SO_2_ in the distal plume (~4 to 6 km from the vent) were determined daily using two fixed scanning ScanDOAS stations (see Materials and Methods; Fig. 2) located near the UV camera and on the coast at Baliau. In addition, SO_2_ flux measurements were made from two boat traverses on 20 May 2019 with a zenith-pointing MobileDOAS unit (see Materials and Methods) and a UAS traverse on 26 May 2019 with a compact MobileDOAS unit. Combining the results from all techniques, DOAS measurements throughout the observation period yield SO_2_ fluxes that range from 2590 ± [1520, 860] to 5200 ± [660, 180] tons day^−1^, with a mean of 4120 ± [1780, 810] tons day^−1^ (Fig. 4E and Table 2). DOAS measurements are not explicitly corrected for dilution; instead, it is included within upper uncertainty bounds, based on previous modeling results (*59*) and a comparison with UAS-derived data (see Discussion).

Wind speeds at plume altitude varied between 1 and 6 m s^−1^ during the week but remained relatively stable over time scales of several hours (Fig. 4D). As direct plume speed measurements could not be made continuously, the time series was complemented with modeled wind speeds at 2000 m above the summit altitude for the evaluation of ScanDOAS data (ERA5 model of European Centre for Medium-Range Weather Forecasts; updated hourly, 31-km horizontal resolution, 137 vertical levels; see Materials and Methods). This approach introduces a random error that is reduced by averaging multiple scans over the measurement period, assuming that the SO_2_ flux does not vary over this time scale. This plume altitude was selected by comparison of plume transport directions from satellite images with vertical wind profiles, supported by ground-based and aerial observations where available. Generally, we observe good agreement between direct wind speed measurements and modeled wind speeds (Fig. 4D), but direct wind measurements using UAS drift speeds at the time of DOAS traverses were used to derive SO_2_ emission rates wherever possible. Further, under conditions of strong vertical wind shear, the injection of two distinct plumes to different altitudes resulted in two contrasting directions of plume dispersion, also with potentially different plume speeds (e.g., Fig. 1B). The ScanDOAS network was often only able to observe one of the plumes—generally the low-altitude plume from the Main Crater—despite additional constraints on plume geometry provided by the two linked systems. Although it is possible to visually inspect the actual scan for each data point, it is difficult to ensure that only data covering plumes from both summit vents are taken forward in the calculation, thus leading to an overall underestimation of the total flux.

### Satellite-based constraints on SO_2_ emission

**Fig. 5:**
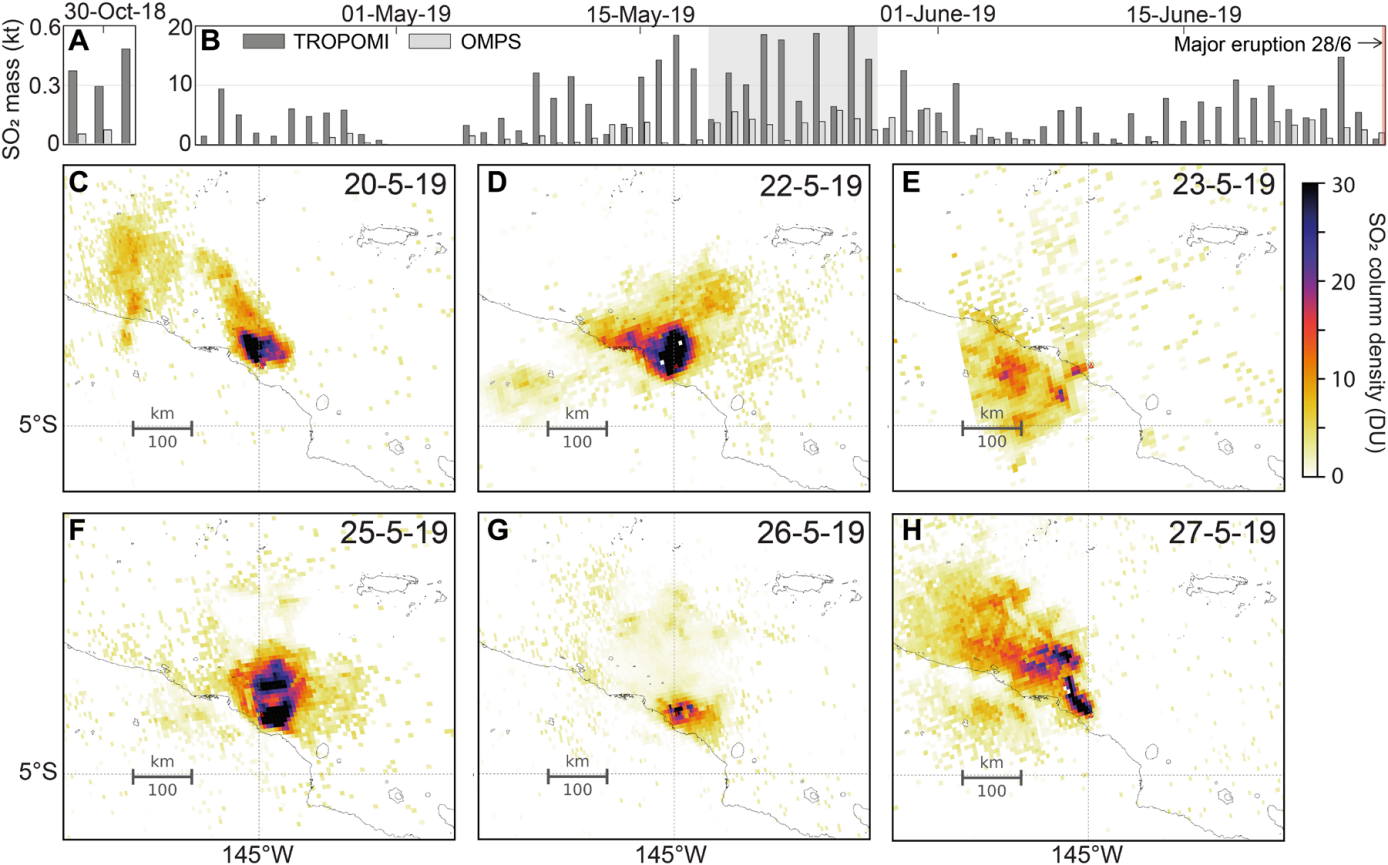
Satellite retrievals of SO_2_ mass loadings. Measurements from TROPOMI and OMPS, interpolated to a plume altitude of 3 km, are shown for the periods (**A**) 29 to 30 October 2018 and (**B**) 26 April to 26 June 2019 and interpolated to a plume altitude of 3 km. Note the different y-axis scales. Gray shaded regions highlight when field measurements are available. Uncertainties are reported in table S3. (**C** to **H**) Maps of SO_2_ column density for the campaign period 20 to 27 May 2019. Wind direction varied on time scales of hours to days, sometimes resulting in the appearance of two distinct plume directions in a single TROPOMI scene. Color scale is in Dobson units (DU), proportional to the number of molecules in a square centimeter of atmosphere. If all the SO_2_ in a column of atmosphere was compressed into a flat layer at standard temperature and pressure, one DU would be 0.01 mm thick and would contain 0.0285 g m^−2^ of SO_2_. Black pixels indicate >30 DU.

Column densities of SO_2_ (where each pixel represents the integrated concentration of SO_2_ through a profile through the atmosphere) were measured by (i) the Tropospheric Ozone Monitoring Instrument (TROPOMI), which overpasses Manam at approximately 04:30 UTC (13:30 LT) each day (*60*), and (ii) the Ozone Mapping and Profiler Suite (OMPS), which has a similar overpass time. TROPOMI column densities were interpolated to a measurement altitude of 3 km and translated into SO_2_ mass loadings by integrating over the area shown in Fig. 5. The OMPS SO_2_ retrievals specifically assume a SO_2_ vertical profile with a center of mass altitude (CMA) of 3 km (i.e., no interpolation is needed); SO_2_ mass loadings were calculated by integrating all OMPS pixels containing >0.3 DU (Dobson units) SO_2_ in the area shown in Fig. 5. Full details of the retrieval approach are given in Materials and Methods. SO_2_ mass loadings during the field campaign in May 2019 are elevated significantly compared to October 2018 (Fig. 5A and figs. S5 and S6). During 29 to 31 October, SO_2_ emissions were barely detectable by either TROPOMI or OMPS with total mass loadings of 0.3 to 0.6 kt SO_2_ (TROPOMI) and <0.1 kt SO_2_ (OMPS) in each scene. These masses are an order of magnitude lower than the mass loadings of 2 to 20 kt SO_2_ retrieved in May 2019. We note that TROPOMI retrievals yield SO_2_ column densities significantly in excess of those derived from OMPS (Fig. 5 and fig. S6).

Maps of interpolated SO_2_ column density during 20 to 27 May 2019 indicate that the plume was not efficiently transported downwind and, instead, remained concentrated in a wide cloud over the island (Fig. 5, D and F). This observation is supported by UAS measurements of low wind speed made at plume altitude (typically 1 to 2 m s^−1^ with a single measurement reaching 6 m s^−1^; Fig. 4D). Figure 5 highlights considerable variability in the direction of plume transport on hourly to daily time scales. For example, column densities measured on 22 May 2019 show elevated values downwind of Manam in two distinct plume directions: NW and NE. This result is similarly supported by ground-based observations, which document a progressive shift in wind direction from NE through NW throughout the morning before the overpass.

A clear image of the Manam plume was acquired during a single overpass of the multiband Advanced Spaceborne Thermal Emission and Reflection Radiometer (ASTER) satellite sensor on 22 May 2019 (fig. S7). Using the 8.6-μm SO_2_ absorption feature in the thermal infrared region, SO_2_ mass loadings can be retrieved with a spatial resolution of 90 m by 90 m (*61*, *62*). A downwind transect of width 17 km, of which the plume width is approximately 14 km, yielded peak SO_2_ mass loadings of ~6 g m^−2^ (equivalent to ~213 DU). The optimal detection limit for SO_2_ of 10 to 20 DU meant that the dilute margins of the plume were not adequately captured, and therefore, the derived SO_2_ mass loading should be considered a lower bound. Considering a wind speed of 2.2 m s^−1^ [the average wind speed measured at plume altitude (UAS and model winds) within a few hours of the overpass; Fig. 4], we derive an SO_2_ flux of 6410 ± 1920 tons day^−1^ [±25% (*62*) to 30% (*61*)].

### Carbon isotope composition (δ^13^C-CO_2_)

**Fig. 6:**
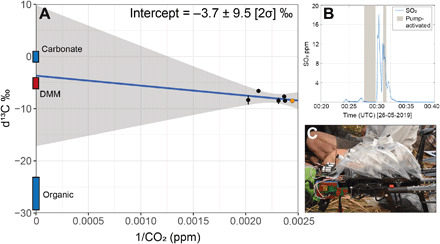
Carbon isotope composition of Manam volcanic gas plume. (**A**) Isotopic composition and CO_2_ concentration of samples collected by UAS in the plume emanating from the Southern Crater (black circles) and in clean ocean air (orange circle), extrapolated to 100% CO_2_ by least squares linear regression (blue line). The gray shaded region shows the 95% confidence bounds on the regression. Uncertainties on the measurements are smaller than the symbol size unless shown. (**B**) The timing of bag sampling (“pump-activated”) shown relative to SO_2_ concentrations measured by co-located Multi-GAS instrument on 26 May 2019. Only the second pump activation was triggered within dense plume conditions (>5 ppm SO_2_). (**C**) Sampling apparatus mounted on the multirotor UAS, comprising four Tedlar bags connected in series with the Multi-GAS and its pump. Image credit: T. Fischer.

We collected a suite of dilute gas samples by multirotor UAS from within the high-altitude plume from the Southern Crater on 26 May 2019. Although very dilute, these samples [421 to 494 parts per million volume (ppmv) CO_2_, δ^13^C-CO_2_ −8.49 to −6.59 per mil (‰)] define one end of a mixing line from a clean ocean air background (409 ± 0.02 ppmv CO_2_, δ^13^C-CO_2_ −8.5 ± 0.1‰) toward that of the magmatic CO_2_ composition (Fig. 6, where δ^13^C is the deviation of the ratio ^13^C/^12^C relative to that of Pee Dee belemnite). The carbon isotope composition remains unconstrained at high CO_2_ concentration, and consequently, extrapolation of this mixing line to 100% magmatic CO_2_ cannot be considered robust. However, for illustrative purposes, extrapolation of this mixing line would suggest a δ^13^C-CO_2_ of −4‰ (±9.5‰; 95% confidence limits) for the magmatic source. Fundamentally, these data demonstrate that near–real-time retrieval and field analysis of plume samples for carbon isotope measurements are not only feasible but also achievable at long range; the key advance required is to ensure sampling of the plume where gas concentrations are highest. Full details of our aerial sampling technique and analytical procedures are given in Materials and Methods.

## DISCUSSION

### Uncertainties associated with ground-based and aerial SO_2_ flux measurements

We present a comprehensive time series of SO_2_ flux measurements for Manam, determined using multiple independent techniques in parallel (Fig. 4). These data offer a rare opportunity to consider the strengths and limitations of contrasting techniques for the case of a complex plume under nonideal atmospheric conditions, which, despite not usually being the subject of instrumental comparison, is a relatively common situation encountered at strongly degassing volcanoes in the tropics. To reconcile the SO_2_ flux estimates derived from the various methods, we first consider the main factors contributing to uncertainty in each of the measurements.

First, the low wind speeds in the horizontally dispersing plume, together with the variability in direction and altitude, introduced considerable uncertainty associated with convolving a plume speed to ICAs of SO_2_. DOAS techniques derive ICAs from near-vertical cross sections through the plume at distances of 4 to 6 km from the vent. Using an average wind speed of 2 m s^−1^ in the horizontally dispersing plume and 12 m s^−1^ in the ~1-km vertically rising region, this implies a plume age of ~35 to 50 min at the point of DOAS measurement. At these distances, low horizontal wind speeds led to a broad, dispersing plume occasionally more than several kilometers in width; this is a challenging geometry for DOAS, which requires complete traverses/scans through the plume with clear sky background on either side. When plumes are very wide, either full traverses/scans are not achievable or the time taken to do them renders the measurement highly uncertain (this was the case for two unsuccessful boat traverses attempted on 21 May 2019). UV camera images, in contrast, are relatively unaffected by downwind plume dispersion as measurements are focused on the plume immediately above the vent, where thermal buoyancy dominates plume transport dynamics.

Second, the multiple gas emission sources at the summit introduce uncertainty associated with incomplete plume coverage. Under conditions of vertical wind shear, the injection of two distinct plumes to different altitudes resulted in two contrasting directions of plume dispersion, as well as potentially different plume speeds (e.g., Fig. 1B). As described in Results, both techniques struggled to provide full coverage for the diverging emission sources, leading to an overall underestimation of the total flux that is difficult to quantify with the available data.

Last, atmospheric scattering of sunlight in the atmosphere between the DOAS instrument and the gas plume causes a “dilution” of the retrieved gas column (*57*, *63*, *64*). Light dilution therefore introduces a systematic underestimation of integrated SO_2_ column amounts, the effect of which becomes magnified as the plume-to-instrument distance increases (among other factors including atmospheric turbidity) (*57*), and is therefore particularly significant at long viewing distances of 4 to 6 km, such as for Manam. UV cameras view at a relatively low angle across several kilometers of the atmosphere and into the densest part of the plume where radiative transfer effects, as well as nonlinearity in SO_2_ absorption, are most significant. Correcting UV camera data for light dilution increases the derived flux by 74 to 160% (see Results), with the correction process associated with its own uncertainties. In reality, part of the underestimation attributed to light dilution is caused by a nonlinearity between the measured apparent absorption and the column density at high optical densities, when the spectral resolution of the instrument is insufficient to fully resolve the absorption bands. In contrast, DOAS measurements are not corrected explicitly, and it is instead included within upper uncertainty bounds (see Results); such differences in postprocessing must be accounted for when reconciling fluxes obtained from multiple techniques. We note that in highly condensed plumes, such as that observed at Manam, internal scattering within the gas plume itself may cause amplification of the total signal, leading to an overestimation of the total column amount of SO_2_, which is not explicitly corrected for by either technique.

Comparing co-acquired SO_2_ fluxes measured on 27 May 2019 by fixed ground-based ScanDOAS and aerial UAS-mounted MobileDOAS, we find that the MobileDOAS traverse yields an emission rate ~20% higher than that acquired by the ScanDOAS instrument operating at about the same time. Considering the sources of uncertainty in DOAS measurements described above, we conclude that the SO_2_ emission rate measured by this UAS traverse is likely to be the more accurate throughout the field campaign. Crucially, this measurement was accompanied by a co-located plume speed measurement at plume altitude using the UAS drift method (6 m s^−1^), reducing the uncertainty associated with using either modeled wind speeds at an assumed altitude (2.5 ± 0.3 m s^−1^), or from an in situ measurement made at a different time or place. Further, the positional flexibility gained by traversing the spectrometer beneath the plume at 1 km asl ensured that the complete plume was covered, free from obstruction at low-scan angles that may affect the ScanDOAS station for certain plume directions. Fundamentally, the elevated measurement position of the traverse would also be expected to reduce the influence of light dilution from atmospheric scattering by decreasing the total distance between the gas plume and the spectrometer.

Overall, despite the significant sources of uncertainty, average SO_2_ fluxes from UV camera and DOAS measurements show reasonable agreement within error. The absolute magnitude of derived SO_2_ emission rates are associated with large, asymmetrical uncertainties (see Materials and Methods), and this propagates to similarly skewed uncertainty bounds on average campaign fluxes (Fig. 4). While DOAS measurements are likely systematically underestimating the total flux due to light dilution, UV camera data are theoretically overestimating owing to the uncorrected effect of in-plume multiple scattering and uncertainties associated with the applied light dilution correction. However, obscuration of the plume and/or incomplete coverage introduces unquantifiable uncertainties on all flux measurements for all but the first day of UV camera measurements on 20 May 2019. It is potentially significant that the derived flux for this day is substantially elevated compared to subsequent acquisition intervals, but we cannot exclude natural variability in the daily SO_2_ emission rate.

### Tracing the carbon flux and source

The style and intensity of volcanic activity remained relatively stable throughout the period of observation; we therefore consider average values based on repeated measurements to be representative of the time-averaged gas composition and flux between 20 and 27 May 2019. Our data show an average gas composition of 91.9% H_2_O, 4.2% CO_2_, and 3.9% SO_2_, typical of high-temperature arc volcanic gases (*6*, *54*). By multiplying each measured CO_2_/SO_2_ mass ratio by the average of all SO_2_ flux measurements made on the same day, we determine the associated CO_2_ flux (Table 1). From these flux estimates, we derive an average CO_2_ emission rate of 3760 ± [600, 310] tons day^−1^. In contrast, the gas composition measured from Main Crater emissions in October 2018 is more water rich: 98.7% H_2_O, 0.7% CO_2_, and 0.6% SO_2_. From these data alone, we are unable to calculate a CO_2_ emission rate for October 2018, as we lack an independent constraint on SO_2_ flux for this period. However, given the much reduced SO_2_ mass loadings (by an order of magnitude) indicated by satellite mass loadings (fig. S5), we suggest that the SO_2_ emission rate was reduced significantly compared to May 2019. The similarity in CO_2_/S_T_ ratios measured during both campaigns suggests that the CO_2_ emission rate may be expected to scale proportionally with changes in total SO_2_ flux, and therefore, the CO_2_ flux would also be proportionally lower in October; however, we do not propose that this assumption holds universally.

The October 2018 campaign followed a period of heightened activity, beginning with a major explosive eruption on 26 August and culminating in a phase of lava extrusion and ash emission in late September to early October. In contrast, measurements in late May 2019 preceded a major explosive eruption on 28 June. Although the timing of measurements relative to eruptive activity is different between October and May, similar CO_2_/S ratios require degassing over a similar range of pressures; the difference in gas emission rate that we infer from satellite SO_2_ mass loadings and visual observations must therefore reflect a change in either the deep gas supply or the permeability of the shallow conduit magma. Satellite SO_2_ mass loadings reduced to very low values (comparable to October) between 3 and 15 June before reaching another maximum on 18 June and then declining slowly once again (Fig. 5). This variability, reflected in both TROPOMI and OMPS time series, demonstrates that precursory activity ahead of the major eruption on 28 June cannot be described by a simple escalation of volatile emission rates through time—assuming that atmospheric conditions and therefore SO_2_ lifetimes remained comparable.

Interpretations of gas compositions in a global context rely on the assumption that the emitted gas phase is representative of the volatile content of the parental magma. CO_2_ and S are known to have different solubilities in magmas, and therefore, shallow magma ascent and decompression should be tracked by decreasing CO_2_/S_T_ ratios and increasing SO_2_ flux. However, observations of CO_2_-rich gas emissions before large eruptions—as reported at several well-monitored open-vent mafic arc volcanoes (*36*, *65*–*67*)—require rapid, disequilibrium gas ascent and thus relatively transient excursions in the long-term volatile budget. Poor correlation between compiled CO_2_/S_T_ ratios and corresponding long-term average SO_2_ fluxes suggests that tectonic setting, and more specifically the subducted sediment contribution to the volatile content of the parental magma, exerts a stronger control on the time-averaged CO_2_/S_T_ than the pressure-dependent degassing mechanisms that fractionate CO_2_ from S (*11*). Passive degassing at open-vent volcanoes during intereruptive periods can generally be reproduced by models of closed-system degassing, whereby the gas phase remains coupled to the melt throughout ascent, before segregating at relatively shallow depths beneath the surface (*67*–*69*); under this regime, the emitted gas composition represents the cumulative gas phase degassed over a range of pressures (*11*). Aerial observations at Manam in May 2019 clearly show an open-vent condition, with magma present at shallow levels in the conduit (Fig. 1). Although low-pressure degassing of a shallow, stagnant body of magma would yield a gas phase dominated by SO_2_ (and thus a CO_2_/S_T_ ratio much lower than that of the parent magma), this scenario is inconsistent with the large SO_2_ fluxes of ~5000 tons day^−1^ observed. The volume of magma degassing magma required to sustain an SO_2_ flux of this magnitude each day is huge: 0.4 to 0.7 km^3^ day^−1^, based on an undegassed sulfur content of 0.2 ± 0.02 weight % and varying vesicularity between 0 and 30% (fig. S8; see the Supplementary Materials for details of the model calculation and parameter ranges). It is difficult to explain such strong SO_2_ emission without sustained magma convection supplying volatiles from depth, although this raises interesting questions about the ultimate fate of the degassed, nonerupted magma.

The CO_2_/S_T_ molar ratio of the emitted gas from Manam is significantly lower than that predicted based on trace element relationships (*11*). With a measured CO_2_/S_T_ ratio of 1.07 ± 0.06, Manam sits firmly within the group 1 classification of volcanoes (*6*, *11*) and thus toward a carbon-poor magmatic volatile end-member composition. Group 1 volcanoes are characterized by gas CO_2_/S_T_ ratios <2 and low whole-rock Ba/La ratios (<50) and globally are associated with subduction of carbon-poor sediments such as terrigenous material or altered oceanic crust (or by carbon-rich material on the slab failing to enter the magma source region, e.g., by being scraped off during shallow subduction). Limited whole-rock data available for Manam indicate a Ba/La ratio of 30 to 60 (*n* = 8), consistent with, but not solely diagnostic of, a group 1 association. However, on the basis of assumptions made about the regional carbonate compensation depth, Aiuppa *et al.* (*11*) model Manam’s gas composition according to the global CO_2_/S_T_ versus Ba/La relationship for group 2 volcanoes, where subducted carbonate sediments supply carbon-rich fluids to the magma source region. Consequently, Manam was predicted to have a source CO_2_/S_T_ signature >2, which we show is likely to be an overestimation.

Considering modern geophysical reconstructions of the rather unique tectonic regime in which Manam is situated (Fig. 1A), the observed discordance with global geochemical trends is perhaps not that unexpected. Following arc-continent collision and partial obduction of the Adelbert-Finisterre Terrane over the leading edge of the Australian continental crust, the Western Bismarck arc is no longer a site of active subduction (*43*–*46*) (Fig. 1A). Regional convergence is now accommodated along the Ramu-Markham fault zone leaving a hanging remnant slab beneath the southern portion of the arc, the margins of which are outlined by distinct gravity and seismic signatures (*46*, *47*). Any carbonate sediments entering the Western Bismarck trench during closure of the Solomon Sea are likely long melted and replaced by carbon-poor sediment addition following terrane accretion, potentially sourced from the underthrust continental crust (*45*, *46*, *70*). In general, the available geochemical data from West Bismarck lavas are not consistent with a substantial addition of material other than the mantle source. The low Ti contents of Manam lavas are indicative of a highly depleted mantle source region (*42*), and Pb isotope and incompatible trace element data (*45*) suggest the addition of a limited terrigenous sediment component. In the context of geophysical constraints, petrological signatures are consistent with progressive heating and melting of a remnant slab into a stagnant mantle wedge no longer rejuvenated by corner flow. Returning to the discussion of CO_2_/S_T_ ratios, the lack of active subduction of carbon-rich sediments points to a tectonic regime much more similar to that of other group 1 volcanoes worldwide.

The extrapolated carbon isotope composition δ^13^C-CO_2_ of −4.0 ± 9.5‰ based on magmatic gas emissions from Manam lies within the global mean volcanic gas composition for arc volcanoes of −3.8 to −4.6‰ (*7*). Considering the large uncertainty associated with extrapolation of a mixing line from such dilute samples, we cannot make conclusive statements about the carbon source as, statistically, we cannot distinguish beyond the range of uncertainty between upper mantle carbon (δ^13^C-CO_2_ = −6.5 ± 2.5‰) (*4*) and marine limestone carbonate (δ^13^C-CO_2_ ≈ 0‰). However, the lack of regional subduction of organic-rich sediments, together with the positive trajectory of the mixing line, suggests that a significant contribution from sedimentary organic carbon (δ^13^C-CO_2_ = −30 ± 10‰) is unlikely, consistent with the limited or carbon-poor sediment supply to the trench suggested by low CO_2_/S_T_ gas ratios.

### Bromine chemistry

Measured BrO/SO_2_ ratios are toward the lower bound of observed values for arc volcanoes globally, which range over three orders of magnitude (*71*) from 10^−6^ to 10^−3^ (fig. S9). BrO/SO_2_ ratios in plume emissions vary with changes in eruptive style with explosive activity generally associated with lower BrO/SO_2_, as described for Etna (*72*), Nevado del Ruiz (*73*), and Tungurahua (*74*). These temporal variations are interpreted in the context of different fluid-melt partitioning behavior for bromine relative to sulfur in silicate melts, which fractionates Br from S in the gas phase as a function of degassing depth, among other factors (*72*). Experimental studies suggest that the fluid-melt partition coefficient for bromine (D_Br_^f/m^) depends strongly on the melt composition (using synthetic melts) (*75*, *76*) and temperature, with D_Br_^f/m^ increasing with decreasing temperature (*77*). However, while recent experiments using natural basaltic melts at 100 MPa suggest that bromine is more soluble than sulfur [and therefore degasses at shallow pressures similarly to chlorine (*77*)], empirical observations are best explained in the context of other geophysical parameters if bromine is less soluble than sulfur [and therefore degasses earlier at higher pressures (*72*, *73*, *78*)]. Positive co-variation between CO_2_/SO_2_ and BrO/SO_2_ ratios during changes in lava lake level at Nyiragongo would suggest that BrO behaves similarly to CO_2_, which has a low solubility in silicate melts (*78*). Further experimental work over a range of pressures, melt compositions, and oxidation states are required.

In the context of previous observations that describe reduced BrO/SO_2_ during high-intensity eruptive activity at other volcanoes, the low BrO/SO_2_ ratios [relative to global arc averages (*71*)] measured at Manam may reflect elevated degassing in late May 2019, ahead of the major eruption taking place 1 month later. However, without a low-activity baseline to compare to—and knowing the sensitivity of D_Br_^f/m^ to melt composition—we cannot evaluate whether our measured values are unusually low or high for Manam. Alternatively, the low BrO/SO_2_ ratio may reflect a low halogen content in the Manam plume, due to (i) a halogen-poor parental melt, (ii) a lower D_Br_^f/m^ due to a specific permutation of melt composition and degassing conditions that is not yet constrained experimentally, or (iii) limited transformation of emitted bromine, as HBr, into BrO in the atmosphere. The reactivity of bromine in the plume can be reduced if there are either insufficient amounts of HO_2_ or O_3_, or abundant water vapor, which dilutes the aerosol content of the plume and therefore slows down the “bromine explosion” mechanism (*79*, *80*). As discussed below in relation to SO_2_ lifetimes, the high moisture content of the tropical atmosphere potentially favors the latter explanation, which would lead us to underestimate BrO/SO_2_ ratios. However, without constraints on total bromine emission or the local abundance of HO_2_ or O_3_, we cannot distinguish unambiguously between these potential scenarios.

### Gas emissions from Manam in a global context

In the broader context provided by satellite observations, the measurements presented here from May 2019 were made during a period of elevated SO_2_ emissions relative to the preceding or following months. From TROPOMI retrievals, the total mass of SO_2_ emitted between 20 and 28 May 2019 (108 kt) contributed 32% of the total cumulative mass loading between 20 April and 26 June 2019 (337 kt; 64 days), with daily SO_2_ mass loadings approaching or exceeding 15 kt during four of our eight field days. However, SO_2_ mass loadings from OMPS are only 2 to 10% of those measured by TROPOMI (Fig. 5). The mass loadings derived from TROPOMI and OMPS data during the field campaign co-vary linearly [SO_2_^(OMPS)^/SO_2_^(TROPOMI)^ = 0.27 ± 0.06; fig. S6], whereby the magnitude of the difference between the two sensors increases proportionally with increasing SO_2_ mass. Crudely, if we assume a typical SO_2_ lifetime of 1 to 2 days in the lower troposphere, then a peak mass loading of 15 kt (TROPOMI) would translate to 7500 to 15,000 tons day^−1^, while 6 kt (OMPS) would translate to 3000 to 6000 tons day^−1^. The estimate from TROPOMI exceeds even the upper limit of uncertainty on ground-based measurements of SO_2_ flux. The discrepancy in absolute SO_2_ mass loadings from different satellite platforms merits further discussion that is largely beyond the scope of this paper. Crucially, however, the SO_2_ detected by OMPS between 20 and 28 May also contributed 33% of the cumulative mass loading over the same period. Therefore, despite uncertainty in absolute SO_2_ mass loadings, relative changes through time appear significant.

Considering several sources of uncertainty, if there is relict SO_2_ persisting from previous days, satellite mass loadings (i.e., fresh SO_2_ plus relict SO_2_) can overestimate the total daily emission rates. At Manam, the persistence of SO_2_ in the satellite field of view may have been extended by the variable wind direction and low wind speeds measured at plume altitude (Fig. 4D), leading to accumulations of relict gas over the volcano’s summit (supported by background reference spectra from DOAS measurements; see Materials and Methods). However, high atmospheric water contents in the tropical troposphere could shorten the SO_2_ lifetime by enhancing wet deposition, in which case mass loadings are an underestimate of daily emission rates. Each satellite scene may also contain SO_2_ emissions from additional volcanoes: Kadovar (located 50 km north of Manam) was also erupting during both field campaigns. Because of the lower activity from Manam during the October 2018 field campaign, plumes from Kadovar were clearly visible and contributing to scene mass loadings (fig. S5). During May 2019, the activity level from Manam was considerably higher, and the SO_2_ plumes observed by satellite may therefore be a composite of both volcanoes’ emissions (Fig. 5).

With a CO_2_ flux of 3760 ± [600, 310] tons day^−1^, Manam currently ranks among the strongest volcanic carbon emission sources globally [rank 7th; based on the data compiled by Aiuppa *et al.* (*11*) and rank 5th based on Fischer *et al.* (*12*)]. Although this is within uncertainty of the predicted carbon flux of 2755 ± 1570 tons day^−1^ based on non-volatile trace element relationships and long-term average SO_2_ fluxes measured by satellite (*11*), this agreement is somewhat coincidental as our measurements of elevated SO_2_ emissions are counterbalanced by our lowered CO_2_/S_T_. If emissions were maintained at the measured level over 12 months, the annual flux of CO_2_ would approach 1.4 Mt year^−1^, or ~0.4 Mt C year^−1^ [equivalent to ~3% of the total global outgassing carbon flux estimated to be between 38.7 ± 5.7 (*11*) and 51.3 ± 5.7 Mt year^−1^ (*12*)]. Combining our measured CO_2_/SO_2_ ratio with satellite-derived annual SO_2_ mass loadings between 2005 and 2015 (*16*) suggests that the annual CO_2_ output of Manam may have fluctuated between 0.25 and 0.92 Mt year^−1^. However, we note that annual averages can alias variations in SO_2_ flux on time scales of weeks to months, and therefore, it is likely that our CO_2_ fluxes are not exceptional and that fluxes of a similar magnitude have occurred transiently in the past. Further, given the relationship between gas ratios and degassing pressure and/or open versus closed system degassing conditions (*65*, *69*, *81*, *82*), it is likely that the emitted gas composition is coupled to eruptive style in such a way that CO_2_/SO_2_ ratios do not scale linearly with SO_2_ flux over long time scales.

Our measured volatile fluxes at Manam are substantially in excess of the decadal mean SO_2_ emission rate (*16*) (1484 ± 753 [1σ] tons day^−1^). However, temporal variability in SO_2_ emissions observed at arc volcanoes more generally (*13*, *16*, *83*) indicates that degassing budgets are highly dynamic and should be extrapolated with caution. The difference in satellite SO_2_ mass loadings between October 2018 and May 2019 at Manam (Fig. 5 and fig. S5) provides compelling evidence for this. Long-term average volatile fluxes are inherently biased for those volcanoes characterized by transient, high-flux eruptive periods separated by long periods of repose or low-level passive degassing (e.g., Tavurvur, Rabaul, Papua New Guinea) (*16*). This bias is especially problematic for remote or rarely accessed volcanoes where campaign measurements are infrequent and/or concentrated during periods of heightened activity (*13*).

### Aerial measurements as a new frontier for volcanic gas measurements

High-altitude BVLOS UAS measurements are pushing the frontiers of the current state of the art in volcanological instrumentation. The development of low-cost, high-endurance UAS in tandem with the miniaturization of sensor payloads has opened new avenues for research and monitoring at previously inaccessible active volcanoes. Here, we have presented an integrated approach combining in situ UAS measurements with near-contemporaneous ground-based remote sensing: an approach that has enabled us to derive multi-species gas fluxes that would otherwise not have been possible using established ground-based methods. Manam presents a situation where in situ samples would have been incredibly hazardous to collect; not only would ascent of the steep slopes present considerable risk in practical sense, but with a major eruption taking place only 1 month after our campaign, the potential for rapid escalations in eruptive activity is clear. Further, a lack of baseline monitoring data against which to compare ongoing activity precludes any robust real-time assessment of risk. This risk scenario is not unique to Manam volcano; long-range UAS deployments (2 to 10 km) remove the need to take unreasonable risks to obtain proximal samples where safe approach cannot be evaluated.

From a scientific perspective, the versatility of UAS enables much more flexible experimental designs. We have discussed here how UAS-mounted spectrometer traverses (for the calculation of SO_2_ flux) increase the likelihood of a complete traverse with clear background on either side of the plume, remove issues related to field-of-view obstruction at low-scan angles (e.g., by vegetation or topography), enable near-contemporaneous wind speed measurements at plume altitude, and minimize radiative effects such as light dilution by reducing the distance to plume. Looking to the future, the ability to select sampling distances in a systematic and controlled way, unconstrained by limitations imposed by ground-based access, will present a significant methodological advance for studies of downwind plume chemical reactions, such as halogen chemistry or aerosol gas-particle phase reactions.

Manam is one of many active volcanoes worldwide where an urgent need for monitoring data must be balanced against considerable risk. Volcán de Fuego, Guatemala, for example, produces frequent, subhourly Strombolian explosions that eject large ballistic projectiles over the upper flanks, with larger, more sustained paroxysmal eruptions occurring on time scales of weeks to months, often generating pyroclastic flows. Following a destructive eruption on 3 June 2018, remote geophysical monitoring resources at Fuego were expanded with installation of further seismic and infrasound stations. However, measurements of gas chemistry remain unconstrained, precluding identification of potential precursory changes in CO_2_/SO_2_ or other volatiles that have been observed ahead of several paroxysmal eruptions at mafic open-vent volcanoes elsewhere (*65*–*67*). With steep, precipitous flanks and a summit altitude of 3763 m asl, aerial access at Fuego requires BVLOS operations on a scale even more ambitious than Manam. Further, unlike the persistent open-vent outgassing from Manam, gas emission at Fuego is strongly pulsatory and requires either careful launch timing and an element of luck or greater UAS endurance to allow loiter time. Schellenberg *et al.* (*40*) describe successful interceptions of the proximal plume involving 2 km of vertical ascent and 9 km of horizontal flight with a fixed-wing UAS. However, these flights were achieved with a minimal payload; addition of gas sensing instrumentation would affect flight endurance considerably. Other volcanoes that are known from satellite or remote-sensing measurements to produce persistent SO_2_ emissions and are capable of generating large eruptions but that are almost entirely inaccessible to ground-based proximal sampling include Bagana and Ulawun (Papua New Guinea), Mayon (Philippines), and Sinabung (Indonesia), among others. With summit altitudes in the range of 1800 to 2500 m asl and closest reasonable approach distances of several kilometers, plume measurements at these volcanoes are achievable at the upper limit of current UAS capabilities. These examples present attractive opportunities to expand the application of aerial strategies within a framework of existing ground-based monitoring networks. Popocatepetl (5393 m asl; Mexico) is one of the most prodigious volcanic CO_2_ emitters on Earth and a key location to explore the role of assimilated crustal carbon in emissions budgets (*7*). With continued UAS innovation, instrumented flights capable of intercepting the near-vent plume of high-altitude emitters such as Popocatepetl—requiring more than 3000 m of vertical ascent—may be realizable.

Although providing effective solutions to many of the limitations associated with ground-based or remote-sensing measurements, it is important to highlight that BVLOS UAS-based techniques can also introduce or amplify several sources of uncertainty when applied to volcanic gas emissions. Signal-to-noise ratios in gas composition datasets can be lower as a result of the reduced plume sampling times imposed by power restrictions on flight time (typically 5 to 30 min for multirotor platforms and 20 to 50 min for fixed-wing aircraft). This is most critical for long-range flights where sampling time is only a fraction of the total travel time. Shorter sampling windows also prevent quantification of temporal variability on time scales longer than several minutes (*33*, *84*). Combustion-powered UAS with an endurance in excess of 50 min can overcome this limitation (*39*) but at the same time introduce the potential for CO_2_ and H_2_ contamination from combustion gases, as experienced using conventional manned aircraft (*27*). From a legal standpoint, platforms containing combustion engines typically fall within larger classes of UAS and therefore require more stringent permissions and licenses to fly.

Differences in sensor response times between gas species introduce uncertainty for derived gas ratios for ground-based measurements (*33*, *85*), and this effect is amplified for aerial instruments. Response times, in the form of the T90 rise time (the time required for the sensor to equilibrate to 90%, when exposed to a step change in concentration), are generally on the order of tens of seconds for electrochemical sensors (e.g., SO_2_) and nondispersive infrared (NDIR) spectrometers (e.g., CO_2_). Fixed-wing platforms, for example, fly at 15 to 25 m s^−1^; therefore, each measurement at 1 Hz represents a spatially averaged concentration with truncated peak amplitudes—the effect is akin to applying a low-pass filter to the true input signal. For narrow plumes less than a few hundred meters across, sensors may not have time to approach equilibrium. Similarly, sensor delays can introduce significant location errors on measured concentrations when traversing the plume at speed, as delays of even 5 s can result in lateral positional offsets of up to 100 m in the direction of travel. For multirotor platforms, the possibility to take samples while hovering inside the plume comes as an advantage but only when the distance to reach the plume is not too long. Typical ascent speeds are 5 m s^−1^, and horizontal speeds are 10 m s^−1^. To reach a plume at a distance of 4 km, the roundtrip would take less than 15 min, so, in principle, there is time to sample the plume for durations well in excess of the sensor response time. However, the required concentration above detection limits is usually only found close to the vent, where the plume may be highly turbulent. High-frequency variations in concentration will not be captured well by slow sensors if further corrections are not applied. Here, we have applied inverse sensor modeling to reproduce the original signal based on a quantitative characterization of sensor-specific filtering properties (see Materials and Methods), and continued implementation of this approach should be a target for future studies. The limitation of response time is eliminated if gas samples are collected in the plume, as was done here for isotopic composition analysis.

Changes in atmospheric pressure (and also temperature) affect both electrochemical and spectroscopic sensors, with measurements acquired at pressures lower than calibration resulting in systematic shifts in detected gas concentrations. Although often internally accounted for, absolute pressure changes or rates of change outside of calibrated ranges can introduce uncompensated nonlinear effects. As we have shown, UAS now enable flights with altitude changes in excess of 2000 m, and therefore, the effects of varying barometric pressure are becoming increasingly significant. Plume traverses at constant altitude, with significant equilibration time on either side, provide a workable solution for the determination of gas ratios (where relative change is more critical than absolute), as used here. However, this is not always practicable and therefore demands an improved characterization of how different sensors respond when ambient pressure and temperature are changing rapidly.

UAS platforms generate considerable air turbulence in the vicinity of the propellers. This is most strongly manifest in multirotor platforms but may still require consideration for fixed-wing aircraft depending on where the inlet tube passing gas to the sensor payload is positioned. The magnitude and scale of the turbulent eddies are not equal in all directions and extend to between 0.5 and >1.5 m away from the UAS depending on direction and the vehicle dimensions (*86*, *87*). Each aerial Multi-GAS measurement therefore represents a spatially averaged gas concentration over a poorly constrained plume volume and thus necessitates the assumption of plume homogeneity over this length scale. While this assumption is likely to be valid for gas sampling, the turbulence envelope may have significant implications for size-fractionation during particulate sampling (i.e., volcanic ash and aerosols). This assumption imposes a lower limit on the spatial resolution of measurements, with implications for the independent characterization of closely spaced emission sources.

Increased automation of sampling flights and the development of intelligent onboard “plume finding” algorithms that make use of real-time data streams will improve the repeatability and precision of UAS-based aerial strategies for gas measurements. These developments in automation will also expedite deployment of UAS in a hazard monitoring capacity.

### Conclusions

Improving our ability to measure and monitor volcanic plumes remotely will have transformative consequences for the quantification of global volatile budgets and for our understanding of volcanic plume dynamics and chemistry more generally. Further, these advances can be translated into tangible monitoring strategies that will, in the future, assist in the identification of precursory changes in volcanic activity at inaccessible volcanic systems. Using novel UAS technology, we present a comprehensive characterization of the volcanic gas composition, isotope signature, and multispecies gas fluxes at Manam, one of the most active volcanoes in Papua New Guinea and a major volcanic emission source on a global scale. We test predicted carbon fluxes based on trace element relationships and find that Manam is a major (rank 5th to 7th) contributor to global volcanic outgassing, with fluxes of 3760 ± [600, 310] tons day^−1^ CO_2_ and 5150 ± [730, 340] tons day^−1^ SO_2_. A relatively low CO_2_/S_T_ signature of 1.07 ± 0.06 suggests a limited or carbon-poor (e.g., terrigenous) sediment supply to the subduction zone, in contrast to previous estimates predicted on the basis of a carbonate-rich equatorial setting. We suggest that this discrepancy may be due to regional tectonics where, following arc-continent collision, the sub-arc region beneath the West Bismarck Arc is no longer the site of active subduction. Instead, most of the emitted carbon may be derived from the depleted upper mantle.

We evaluate the limitations and advantages brought by new aerial sensor-platform solutions and highlight several areas of future research, including but not limited to (i) sensor response characterization to enhance signal-to-noise during short plume traverses, (ii) pressure and temperature change tolerance of gas sensors during rapid fluctuations, and (iii) propeller turbulence envelope and the implications for representative sample collection. Tropical volcanic plumes in strongly convective atmosphere present challenges for ground-based remote sensing. We discuss the associated sources of uncertainty in detail and ultimately demonstrate that aerial measurement strategies can reduce or mitigate against many of these contributing factors. Interpreting our measurements in the context of long-term observations of SO_2_ mass loadings/fluxes from satellite remote sensing, we highlight a precedent for substantial changes in volatile emission rate on both weekly to monthly and decadal time scales, supporting a growing collective of similar observations from other volcanoes globally. We therefore emphasize the need to extrapolate campaign-based measurements with caution and to instead account for temporal fluctuations in volatile emission rates in future estimates of global fluxes.

## MATERIALS AND METHODS

### Unoccupied aerial systems

Aerial measurements were made using both fixed-wing and multirotor type platforms (fig. S3). Permissions for BVLOS operations were obtained from the Civil Aviation Safety Authority of Papua New Guinea. During field campaigns on 29 to 31 October 2018 and 20 to 27 May 2019, an instrumented fixed-wing aircraft acquired visual observations of the summit region and in situ measurements of plume composition. The vehicle was custom built at the University of Bristol based on the “Titan” twin-propeller V-tail airframe (Skywalker, China). The aircraft has a wingspan of 2.1 m and a takeoff weight of 8.5 kg (including ~1-kg payload). The design of this UAS was advantageous because it could be hand-launched and recovered by parachute from the summit of the Godagi cone located in the north of the island (200 m asl; Fig. 2). Power was provided by a 12.7-Ah, 6S 22.2 lithium polymer (LiPo) battery, giving an approximate flight duration of 25 to 35 min depending on altitude and airspeed requirements (nominally 2100 m above takeoff and 18 m s^−1^).

The Titan UAS featured a full autopilot computer with supporting sensors (Global Navigation Satellite System, barometric altitude, airspeed indicator, and inertial measurement unit). Three wireless links were used to interact with the vehicle during flight: a pilot safety link (433 MHz), a bidirectional telemetry modem (868 MHz), and a first person view (FPV) video stream (2.4 GHz). The pilot safety link was used to initialize automated flight paths and for periods of manual control. The bidirectional telemetry modem was used to monitor flight statistics (such as battery consumption), to issue updated commands to the autopilot, and to relay live gas concentration measurements to the ground station.

Plume interception required flights with an altitude gain of 2100 m (above takeoff altitude) and >6 km of horizontal traverse. BVLOS flight operations included both automated and manual flight segments. Preprogrammed waypoint paths were used for takeoff and ascent/descent to/from estimated plume altitude, based on visual observations and coordinates taken from a high-resolution topography model (WorldDEM, Airbus Space and Defence). Plume traverses at constant altitude were either automated or manually piloted using the FPV video stream to ensure direct interception of the plume. Correct positioning was assessed on the basis of a live data stream of gas concentrations. The UAS was manually piloted during alignment of the aircraft with the landing zone and triggering of the parachute deployment.

A multirotor UAS, in the form of a Y-shaped counter-rotating hexacopter (model “Micro,” SkyEye Technologies), was also used during the campaign between 20 and 27 May 2019. Using modular payloads, this UAS acquired in situ measurements of gas composition and wind speed, collected plume samples, and obtained spectroscopic measurements for remote sensing of SO_2_ flux. The aircraft has dimensions of 80 cm by 20 cm by 23 cm (*D* × *H* × *W*), a takeoff weight of <8 kg (including 1-kg payload and batteries) and is completely foldable for field portability. The onboard navigation system is based on open-source Pixhawk technology. Power was provided by two 6S-4P LiPo batteries, yielding a total capacity of 20 Ah. For typical measurement scenarios, this results in typical flight times of 35 min. Two independent radio links were used for operation of the multirotor, one switchable link at 2.4 GHz/900 MHz for pilot safety and another link at 433 MHz for measurement data. Each modular payload was mounted on top of the multirotor and used the same radio link and power from the UAS batteries.

Multirotor flight paths were planned on the basis of a priori information on plume position from the ground-based spectrometer systems (see below). The UAS was flown manually using QGroundControl software. Real-time data on gas concentrations were used to aid positioning of the UAS and to inform the activation of the gas sampling system. Wind speed measurements were made by disabling Global Positioning System (GPS) positioning and measuring the passive drift speed of the UAS. Descent from altitude was performed using energy-saving aerobatic maneuvers whenever possible to maximize endurance.

### Gas composition

Concentrations of CO_2_, SO_2_, and H_2_S in the volcanic plume were measured using two miniaturized pumped Multi-GAS (*88*, *89*) mounted on either fixed-wing or multirotor UAS platforms.

An aerial Multi-GAS from the University of Palermo-INGV was mounted inside the central fuselage of the Titan fixed-wing aircraft described above. Air was sampled through a 1-μm particle filter exposed to ambient air, at a pump rate of 1.0 liter min^−1^. Data were logged at 1 Hz. SO_2_ and H_2_S electrochemical sensors (T3ST/F-TD2G-1A and T3H-TC4E-1A; both City Technology) were calibrated for 0 to 200 and 0 to 50 ppmv, respectively, with an accuracy of ±2% and a resolution of 0.1 ppmv. An NDIR spectrometer (Microsensorik Smartgas Modul Premium2) was calibrated for 0 to 5000 ppmv CO_2_ with an accuracy of ±2% and a resolution of 1 ppmv. The unit was shielded from radio frequency interference from the UAS transmission system using a foil bag. Pressure (±1 hPa), temperature (±0.5°C), and relative humidity (±3%) were also measured at 1 Hz using a Bluedot BME280 sensor exposed to ambient air. The Multi-GAS was calibrated with standard reference gases at Università di Palermo before the field campaign but unfortunately could not be retrieved for post-campaign recalibration. All sensor data were logged onboard the UAS to a micro-SD card and also telemetered directly to the ground station where it could be visualized in real time. H_2_O concentrations were calculated from records of temperature and relative humidity, using an ambient pressure of 774 mbar at traverse altitude [according to the Arden Buck equations relating the pressure of vapor saturation to temperature for moist air (*90*)].

Measured gas concentration time series were postprocessed using MATLAB and Ratiocalc software (*91*). CO_2_ concentrations were internally compensated for temperature (±0.2% full span per °C). SO_2_ concentrations were corrected for reduced ambient pressure at altitude using the manufacturer-stated compensation of 0.015% signal per mbar. Although a pressure correction for CO_2_ is required for absolute concentrations, barometric pressure was considered constant for the determination of gas ratios (which are derived from relative changes in concentration), as the UAS was flown at constant altitude for the full duration of plume intersections. Volcanogenic (or “excess”) CO_2_ was resolved from atmospheric background by subtracting the intercept of the linear regression between CO_2_ and SO_2_ (i.e., where SO_2_ = 0) from the raw CO_2_ time series. H_2_S concentrations were within the 13% cross-sensitivity of the sensor to SO_2_ (determined during calibration with standard reference gases) and so were considered negligible. For example, although the absolute detection limit of the sensor is 0.1 ppm, the effective detection limit is 0.13 × SO_2_ ppm. Differences in sensor response characteristics were accounted for using a Lucy-Richardson deconvolution algorithm applied to the CO_2_ time series (*92*). The algorithm is initiated using the measured time series and makes use of a sensor model determined empirically from the response of the NDIR to step changes in calibration gas concentration. The sensor model is best described by a windowed integral and can be thought of as an *N*-point moving average applied to the “true” input signal. Laboratory tests identified the sensor to average over approximately 15 s; hence, *N* = 15 since measurements are stored at 1 Hz. The deconvolution has the effect of removing the inherent filtering effect of the sensor; hence, the recovered input signal shows peaks in concentration that are steeper, narrower, and marginally greater in amplitude than the measured signal, while preserving the integrated area beneath the peak.

The Multi-GAS unit from Chalmers University of Technology was used as a modular attachment to the multirotor UAS. The unit was contained inside a shielded aluminum enclosure with power supply decoupling. It uses an Arduino Mega2560 data logger for control of several instruments: a particle-filtered inlet connected to a pump (0.5 liter min^−1^) coupled to a cavity exposed to electrochemical sensors for SO_2_ and H_2_S [Alphasense A4-series, calibrated within 0 to 50 ppm; ±2σ accuracy of 15 and 5 ppb, respectively; and analog-to-digital conversion resolution of 16 bits resulting in precisions of 2 ppb]. The sampling rate was 1 Hz. The flow is then coupled to an NDIR CO_2_ sensor (SmartGAS FlowEvo; 0 to 1000 ppm range, ±1% noise and precision of 1 ppm). Ambient P, T, and Rh were measured using a BME280 (Bosch). Also included within the unit is an electronic tiltmeter, GPS (Adafruit Ultimate), and an ultrasonic resonant anemometer (FT250) for measurement of wind speed (range, 0 to 75 m s^−1^; accuracy, 0.3 m s^−1^; precision, 0.1 m s^−1^) and wind direction (range, 0° to 360°; accuracy, 4°; precision, 1°).

Data from this Multi-GAS unit were analyzed in MATLAB. Raw data were first corrected for pressure, temperature, and time response before multiplication of the signal by calibration constants (obtained from laboratory calibrations at 22°C and 1 atm). Ambient background CO_2_ concentration (when SO_2_ = 0) was subtracted from the measured signal. Molar ratios were calculated by linear regression of a CO_2_-SO_2_ scatterplot, for data points where SO_2_ > 0.5 ppm. Pressure and temperature correction factors were adopted from manufacturer’s datasheets. Time response was corrected by optimizing the cross-correlation of deconvoluted time series (assuming first-order dynamical response) of CO_2_ and SO_2_ with high variability (*93*, *94*).

### UV camera

The emission rate, or flux, of SO_2_ was sensed remotely using DOAS and UV camera imaging techniques. We used a fully autonomous, portable version of the dual UV camera system developed at the Università di Palermo and described in (*58*). The instrument, powered by a 12-V battery, was equipped with two JAI CM-140GE-UV cameras sensible to UV radiation and fitted with two distinct band-pass optical filters (both of 10-nm full width at half maximum) with central wavelengths of 310 nm (strong SO_2_ absorption) and 330 nm (weak SO_2_ absorption). An embedded PC (JETWAY model NF36-2600) was used to command and control operations and to save the acquired images in the internal solid-state drive memory. The instrument was field-deployed on the summit of Godagi cone (Fig. 2) on 20 May 2019 and was manually commanded to operate (at 0.5-Hz rate) from 22:00 UTC (20 May 2019) to 00.30 UTC (21 May 2019). During this time interval, clear sky conditions prevailed, and a noncondensing, vertically rising plume was nicely observed that allowed for the best dataset to be acquired. During 21 to 26 May 2019, the instrument was left as a permanent fixed station to run autonomously (at 0.5-Hz rate) during daily measurement cycles of 4 hours each. The daily acquisition interval (04:00 to 08:00 UTC) was selected to concentrate observations when cloud cover on top of Manam is normally the lowest. This notwithstanding, a thick cloud cover obscured all observations on 24 to 25 May 2019, and only <2 hours of successful imaging of the plume was possible in the remaining days. Even in these conditions, however, only a fraction (possibly half) of the plume may have been intercepted because of partial cloud cover and/or the plume being partially obscured behind the flank of the volcano, some (unquantifiable) underestimation of the SO_2_ flux compared to the manually obtained flux from 20 May 2019.

The acquired images (520 × 676 pixels at 10-bit resolution) were postprocessed using standard techniques (*58*, *95*). Sets of co-acquired images were first combined to obtain sequences of “absorbance” images and then converted into slant column amount (SCA) images using calibrations derived from either calibration cells (20 to 21 May 2019) or a coaligned Ocean-Optics USB2000+ Spectrometer (20 to 26 May 2019) coupled to a telescope of rectangular, vertically oriented field of view (≈0.3° × 14°). Dual calibrations with both cells and spectrometer on 20 to 21 May 2019 showed good agreement within ±5%. Last, a time series of ICAs were obtained for each dataset by integrating the sequences of SCA images along a cross section perpendicular to plume transport. Multiplication of the ICA by the plume speed yielded the SO_2_ flux time series. Plume speeds (uncertainty, ±5%) were derived using the optical flow algorithm of (*96*) to track the motion of plume gas fronts in image sequences (*58*).

We find that uncertainty in radiative transfer (*59*, *64*, *97*) was the largest source of uncertainty. We used the Vulcamera software (*98*) and the methodology of (*57*) to estimate light dilution along the optical path (~4 km) between the plume and the camera and correct for this. We find that light dilution contributed a factor ~74% (underestimation) of the true SO_2_ flux in our best UV camera dataset (20 May 2019) and circa 90 to 160% in the remaining days (when the atmosphere was less transparent and therefore scattering more intense). We corrected for light dilution explicitly according to Campion *et al.* (*57*) and show both corrected and uncorrected data in Fig. 4. We note that a component of the underestimation that we attribute to light dilution is, in reality, caused by nonlinearity between the measured apparent absorption and the column density at high optical densities. No attempt was made to quantify the effect of in-plume scattering, but the condensed nature of the plume (despite the absence of ash and relatively moderate SCAs) may have contributed a +30% (overestimation) uncertainty (*59*). The overall budget of uncertainty from calibration, plume speed, light dilution, and in-plume scattering, added in quadrature, amounts to ±7% (random) and ±[74, 30]% (systematic), where uncertainty is expressed as [upper, lower]. This uncertainty budget becomes ±7% (random) and ±[0, 30]% (systematic) following the explicit correction for light dilution.

### Differential optical absorption spectroscopy

#### Sulfur dioxide flux

SO_2_ concentrations in plume cross sections were measured by UV DOAS (*99*), a technique that quantifies the slant column of SO_2_ in the instrument field of view using scattered sunlight from the sky as a light source. Evaluation was performed in the wavelength range between 310 and 325 nm and included a measured Fraunhofer reference spectrum and absorption cross sections for SO_2_ (293 K), O_3_ (223 K), a synthetic Ring-effect spectrum derived from a high-resolution solar spectrum (*100*) using the software DOASIS, and a fifth-order polynomial to account for broad-band extinction. The evaluation was done using software from the NOVAC collaboration (Github/NOVACProject), using routines described in (*19*). The integrated concentration of SO_2_ in a cross section perpendicular to the plume transport direction was obtained either by traversing beneath the plume (MobileDOAS) or by scanning through 180° from a fixed position (ScanDOAS). The MobileDOAS instrument was mounted on a mobile platform, such as a boat or UAS. A telescope collected light from the zenith direction and measured the total vertical column of SO_2_ as the instrument was moved below the plume, ideally in a direction close to perpendicular to the plume transport direction. The time and position of each spectrum is logged. During the field campaign, MobileDOAS traverses were made from a boat at sea level (21 May 2019) and from a UAS at altitudes up to 1 km asl (27 May 2019). In addition, the two ScanDOAS instruments made measurements from two fixed locations (Fig. 2A). Here, the plume is intersected using an optical scanning device, acquiring measurements of slant columns of SO_2_ across the plume cross section, which are then converted to vertical columns. The ScanDOAS units are housed in a portable backpack box and powered by a foldable solar panel and a compact 12-V battery (*101*). For both techniques, multiplication of the integrated cross-sectional concentration of SO_2_ with the wind speed at plume altitude yields the SO_2_ flux (*18*, *19*).

The main advantage of ScanDOAS over MobileDOAS is that measurements can be made automatically, during daylight hours, with 5- to 10-min time resolution. Further, the use of two or more fixed instruments used simultaneously allows plume height and plume direction to be derived by triangulation, while the traverse method requires an independent constraint on plume height. Last, if the plume is passing directly over the instrument, then simultaneous column density series can be measured in the upwind and downwind directions (at 2-s time resolution) by a dual-spectrometer configuration. By correlating the two time series, plume speed can be derived indirectly.

The main sources of uncertainty for both techniques are errors in plume speed and “dilution” effects due to atmospheric scattering of sunlight along the path between the instrument and the gas plume (*63*). In highly condensed plumes (also when ash is abundant in the plume, not applicable here), scattering effects inside the gas plume may cause severe effects (*59*, *64*, *97*). In this field campaign, the plume speed is an important source of error because of the low average wind speed, with large relative fluctuation. In the MobileDOAS measurements, this error was minimized using near-simultaneous measurements of SO_2_ and wind (by the UAS drift method; see above). In the ScanDOAS measurements, this error due to variation in wind speed is reduced by averaging a large number of SO_2_ measurements over the full day. The effect of “dilution” caused by atmospheric scattering is difficult to quantify. However, from a comparison between close to simultaneous measurements made by a ground-based ScanDOAS instrument and a UAS-mounted MobileDOAS instrument at 1 km above ground, an underestimation of the order of up to 20% is possible (*93*). The error bars for the DOAS techniques, in this campaign, are therefore skewed toward being likely underestimates.

Another source of error was the potential for incomplete plume scans. As can be seen in Fig. 1B, the gas emission is often split into two plumes reaching different altitudes and, thus, may propagate in different directions and with different plume speeds. Care was taken to ensure that the different DOAS instruments covered the full emission during postprocessing. Although it is possible to visually inspect the actual scan for each data point, it is difficult to ensure that only data covering both plumes are used, especially when the divergence angle between the two plume directions is great. Thus, this effect also tends to cause underestimation in the ScanDOAS data shown in Fig. 4. The overall budget of uncertainty from spectroscopy, geometry, radiative transfer, and wind speed, added in quadrature, amounts to ±[52, 39]% (random) and ±[30, 5]% (systematic) for ScanDOAS, ±28% (random) and ±[30, 5]% (systematic) for MobileDOAS boat traverses, and ±[3, 2]% (random) and ±[10, 1]% (systematic) for MobileDOAS UAS traverses.

All scan/traverse data are normalized to a “clean air” reference spectrum, to cancel out common spectral features not related to the measurement. We use the lowest column in a measurement as reference spectrum and thereby obtain slant columns relative to the column of this reference. We note that if the reference spectrum is not clean and contains SO_2_, we would still obtain a valid, but underestimated, emission measurement of a plume in excess of this background. If instead we evaluate all ScanDOAS data from the field campaign using the cleanest reference spectrum from the week (23 May), then we notice a substantial SO_2_ background of 10 to 100 DU for all days. This is in line with, and sometimes exceeding, the background as seen by the satellite. We note that this may explain the higher emission values seen by the satellites, as compared to the ground-based instruments.

#### Bromine oxide

The BrO/SO_2_ ratio in the Manam plume was measured remotely by MAX-DOAS (a variant on the ScanDOAS described above), which uses spectra of scattered solar radiation measured at different elevation angles to derive the vertical distribution of atmospheric trace gases as either total column densities or as relative values in comparison to a background spectrum. An inertial sensor–based attitude compensating (ISA) MAX-DOAS instrument was deployed on Manam. The ISA MAX-DOAS contains an automatic attitude and motion compensation of the elevation angle; thus, no manual adjustment on the measurement site was required. A rotatable prism reflects the light on a lens with a focal length of 75 mm, which focuses the light on a monofiber with a diameter of 400 μm. The fiber is connected to a temperature-stabilized UV spectrometer. The ISA MAX-DOAS features a narrow field of view of about 0.3°. The Avantes ULS2048 ×64 spectrometer covers a wavelength range of 296 to 460 nm with an optical resolution of 0.64 nm. The spectrometer is thermally coupled to a Peltier element and to a heat sink with a fan outside of the MAX-DOAS instrument. Temperature is regulated to within <0.05°C using a temperature controller TSE v1.1 (Envimes, 2013 model) in combination with a temperature sensor placed close to the spectrometer. All components are housed in a single compact waterproof housing, with no external moving parts. The instrument has small dimensions (20 cm by 30 cm by 13 cm plus a telescope quartz glass tube 12 cm long) and weighs approximately 6 kg. It is powered by 12 V and consumes about 2.0 A in normal operation mode. It also features an embedded computer accessible by local area network (LAN) or wireless LAN, and a GPS receiver with a specified position accuracy of ±2.5 m.

From 20 to 26 May 2019, with the exception of 24 May, spectra were acquired from the Godagi cone (co-located with the UV camera; Fig. 2). Viewing direction, telescope elevation angles, and measurement times are summarized in table S1. The spectrometer was temperature-stabilized to 30°C for all measurements. The volcanic plume was usually condensed, and the meteorological conditions were overcast with low wind speeds. The low wind speeds led to a very broad plume, which often precluded a background measurement free of volcanic gas.

All collected spectra were evaluated using WinDoas V2.10 (*102*) to derive slant column densities (SCDs) of BrO and SO_2_. As scattered sunlight was used as the light source, solar Fraunhofer lines had to be removed carefully to enable sensitive measurements of trace species. A plume-free background spectrum from 23 May 2019 was used as the Fraunhofer reference spectrum (FRS) for all days. BrO was evaluated in the wavelength region from 330.6 to 352.75 nm, which includes four BrO absorption bands (*103*). SO_2_ was analyzed between 360 and 390 nm (*104*) and then compared to the SO_2_ SCDs from the BrO evaluation range. As the two SO_2_ SCDs deviated less than 10%, the SO_2_ SCDs derived from the BrO evaluation range were used for further data analysis to minimize the influence of radiative transfer effects on the BrO/SO_2_ ratio. Multiple reference spectra, including BrO, NO_2_ (246 K), HCHO, O_3_ (223 and 246 K), SO_2_, O_4_, a “ring spectrum,” and the FRS, were simultaneously fitted to the measurement spectra using a nonlinear least squares method (*105*) implemented in the evaluation software WinDoas. Broadband structures were removed using a third-order polynomial. BrO/SO_2_ ratios were calculated by a linear regression from a SO_2_-BrO scatterplot, considering the fit errors of both parameters.

### Satellite SO_2_ mass loadings

TROPOMI (*60*, *106*) is a hyperspectral ultraviolet spectrometer, located on the European Space Agency’s Sentinel-5P satellite platform. Sentinel-5P is a polar-orbiting platform, flying in close formation with the A-Train constellation with a local equatorial overpass time of 13:30 (ascending node). This allows for synergistic measurements with instruments on the other A-Train platforms, such as Ozone Monitoring Instrument (OMI) and OMPS. The instrument consists of four spectrometers covering three hyperspectral bands, ranging from the UV to the near-infrared (270 to 495, 657 to 775, and 2305 to 2385 nm), and has a spectral resolution of 0.25 to 0.54 nm. TROPOMI has a swath width of 2600 km and a spatial resolution (nadir) of 3.6 km by 5.5 km (*106*).

This analysis uses the Level 2 Offline SO_2_ dataset [L2__SO2__, version 1.01.07 from the ESA-Copernicus Sentinel-5P Pre-Operation Data Hub (https://s5phub.copernicus.eu/)], providing SO_2_ total column densities (in DU), calculated using averaging kernels that place the SO_2_ at one of three altitudes (1, 7, and 15 km) (*60*, *106*). To correct the SO_2_ concentration to the assumed plume altitude of 2 km (from ground-based observations), a linear interpolation was performed between the values at the three altitude levels. The plume altitude is usually the largest source of error in satellite retrievals, but by using the same altitude as those from the ground-based measurements, we reduce one of the main sources of discrepancy between the datasets. Other error sources come from the instrument (e.g., noise and the slit function) and from the forward model used in the creation of the L2 data [trace gas absorption profile and meteorological clouds (*92*)]. These errors are defined within the dataset and are used to calculate the error ranges. However, since the errors we report do not include the plume height, they should be considered the least stringent bounds of the error range.

We also use SO_2_ column retrievals from National Aeronautics and Space Administration (NASA) OMPS, in polar orbit aboard the Suomi-National Polar-orbiting Partnership satellite since 2011. OMPS is a hyperspectral UV instrument with a spatial resolution at nadir of 50 km by 50 km and high sensitivity to volcanic SO_2_ emissions (*107*). Operational OMPS SO_2_ retrievals [OMPS_NPP_NMSO2_PCA_L2; available from the NASA Earthdata portal (https://earthdata.nasa.gov/)] use a principal components analysis algorithm (*108*, *109*), which yields SO_2_ retrievals with very low background noise. Here, we use the lower tropospheric (TRL) OMPS SO_2_ product that assumes an SO_2_ vertical profile with a CMA of 3 km (i.e., close to the average plume altitude observed at Manam during the field campaign). Note that this obviates the need for SO_2_ column interpolation to the plume altitude, in contrast to the TROPOMI measurements. Daily OMPS SO_2_ mass loadings in the Manam region were calculated by integrating all OMPS pixels containing >0.3 DU SO_2_, where 0.3 DU is approximately equal to the 3σ background noise level in OMPS TRL SO_2_ data at tropical latitudes (*108*). Data from OMI are also included in table S3, with a similar retrieval method to OMPS. However, the data are very incomplete due to the known row anomaly issue and are therefore not discussed in detail here.

### Carbon isotope composition

We collected plume samples for subsequent isotopic analysis using a pumped bag sampling unit (four bags per flight), mounted as a modular unit on the multirotor UAS described above. Each bag was connected to a small rotary pump that was automatically triggered by a timer. The time delay before sampling was set in accordance with the estimated flight time from launch to plume interception. The duration of sample collection was approximately 45 s at an approximate flow rate of 1 liter min^−1^. A valve system was not necessary because the pump also functioned as a valve once pumping stopped. On landing, the valves on the Tedlar bags were sealed. In addition to plume samples, a clean air sample was collected upwind at plume altitude to characterize the carbon isotope composition of ambient air.

The samples were analyzed in the field within hours of collection using a Delta Ray infrared spectrometer, following the analytical procedure described by Fischer and Lopez (*27*). Because of the remoteness of Manam, and the challenges associated with obtaining and transporting calibration and CO_2_-free air gases in Papua New Guinea, we developed an air purification system that used a hand-powered bicycle pump and CO_2_ scrubber, Sulfolime, to produce pressurized (at least 1 bar) CO_2_-free air. This system allowed for the production of essentially unlimited amounts of CO_2_-free air with CO_2_ contents of <0.7 ppm, as measured using the Delta Ray. Our calibration gas was pure CO_2_ obtained from a local distributor. Before analyses, the carbon isotope composition of this gas was unknown, and we therefore collected a sample of this gas to analyze in the Volatiles Laboratory at the University of New Mexico using the Delta Ray and our standard calibration gases. Therefore, we were not able to determine the exact carbon isotope compositions of the samples in the field but were able to obtain relative ratios and abundances compared to our air samples. This information allowed us to evaluate the success of our sampling campaigns while still on the island. We were also able to adjust our pure CO_2_ gas by dilution with CO_2_-free air to the expected concentration of our samples. After analyses in the laboratory at the University of New Mexico of the pure CO_2_ gas from the pressurized gas bottle, we retroactively corrected all our measurements obtained on Manam; these are the data that are reported in table S4.

## Supplementary Material

abb9103_Table_S3.xlsx

abb9103_Table_S2.xlsx

abb9103_SM.pdf

abb9103_Table_S1.xlsx

## SUPPLEMENTARY MATERIALS

Supplementary material for this article is available at http://advances.sciencemag.org/cgi/content/full/6/44/eabb9103/DC1
