# Supplementary material for: Aerial strategies advance volcanic gas measurements at inaccessible, strongly degassing volcanoes
Source: Sci Adv. 2020 Oct 30;6(44):eabb9103. doi: 10.1126/sciadv.abb9103 (PMC7608812; doi:10.1126/sciadv.abb9103)
Supplement: abb9103_SM.pdf [file abb9103_SM.pdf]

Science Advances
AAAS

advances.sciencemag.org/cgi/content/full/6/44/eabb9103/DC1

Supplementary Materials for

Aerial strategies advance volcanic gas measurements at inaccessible, strongly degassing volcanoes

E. J. Liu*, A. Aiuppa, A. Alan, S. Arellano, M. Bitetto, N. Bobrowski, S. Carn, R. Clarke, E. Corrales, J. M. de Moor, J. A. Diaz, M. Edmonds, T. P. Fischer, J. Freer, G. M. Fricke, B. Galle, G. Gerdes, G. Giudice, A. Gutmann, C. Hayer, I. Itikarai, J. Jones, E. Mason, B. T. McCormick Kilbride, K. Mulina, S. Nowicki, K. Rahilly, T. Richardson, J. Rüdiger, C. I. Schipper, I. M. Watson, K. Wood

*Corresponding author. Email: emma.liu@ucl.ac.uk

Published 30 October 2020, *Sci. Adv.* **6**, eabb9103 (2020)
DOI: 10.1126/sciadv.abb9103

The PDF file includes:

Legends for tables S1 to S3
Table S4
Figs. S1 to S9
Calculation of magma volumes required to generate the observed SO_2 flux.

Other Supplementary Material for this manuscript includes the following:

(available at advances.sciencemag.org/cgi/content/full/6/44/eabb9103/DC1)

Tables S1 to S3

Supplementary Materials

Supplementary Files

Table S1 (.xls): Measured gas concentration data (unprocessed and uncorrected for sensor response effects, pressure etc.) used to determine molar gas ratios. Data for Figure 3.

Table S2 (.xls): MAX-DOAS measurements of BrO/SO_2. Data for Figure S4.

Table S3: (.xls) Satellite (TROPOMI, OMPS and OMI) SO_2 mass burdens, interpolated to 2 km and 3 km with uncertainties. Data for Figure 3 and Figure S5, S6.

Table S4: Carbon isotope compositions from plume samples collected on 26 May 2019. Data for Figure 6.

Sample	CO_2	±	$\delta^{13}C$	±	$1/CO_2$	±
Southern Crater_1	421.1	0.23	-8.49	0.89	0.00237	1.32E-06
Southern Crater_2	432.6	0.12	-8.49	0.39	0.00231	6.36E-07
Southern Crater_3	422.7	0.21	-7.66	0.62	0.00237	1.17E-06
Southern Crater_4	494.2	1.05	-8.32	0.16	0.00202	4.31E-06
Southern Crater_5	471.5	1.35	-6.59	0.09	0.00212	6.05E-06
Clean Manam air (over ocean)	409.2	0.02	-8.50	0.10	0.00244	1.42E-07

Additional online materials

For more information on these field measurements, please see the accompanying documentary video "Above and Beyond" produced by Retroscope Media:

https://www.youtube.com/watch?v=H6xMlCrRJyc (20 minutes)

https://vimeo.com/369354180 (3 minutes)

Supplementary Figures

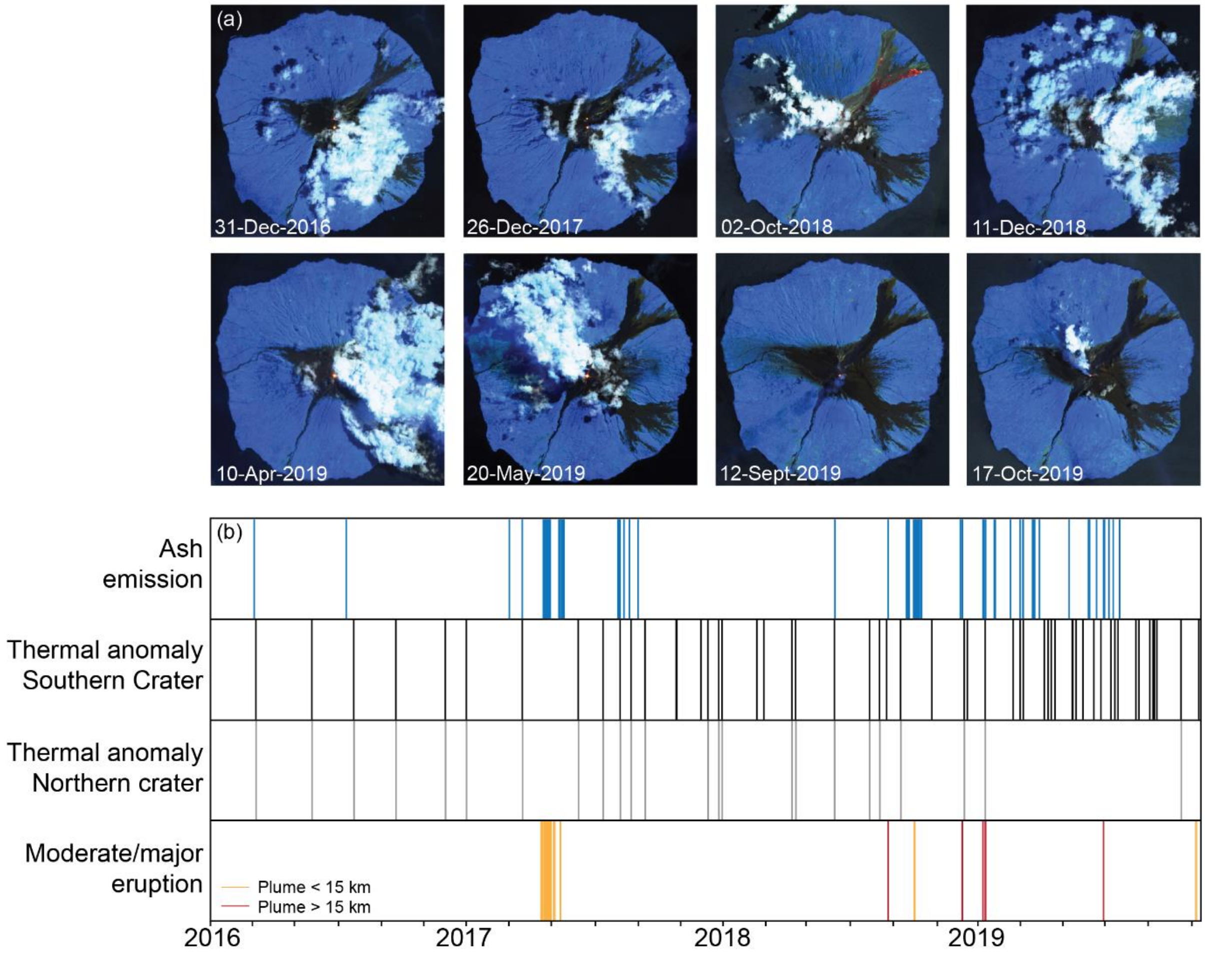

Figure S1: Satellite observations of volcanic activity at Manam, Papua New Guinea, between 1 January 2016 and 30 September 2019. (a) Thermal anomalies were identified at either one or both summit craters by manual inspection of Sentinel-2 L1C imagery (R: 2202.4 nm, band 12; G: 1613.7 nm, band 11; B: 864.7 nm, band 8A) throughout the period of observation. Active lava flows were visible in the northeast valley during the acquisition on 2 October 2018. (b) Summary of volcanic activity, compiled from Sentinel-2 L1C imagery (courtesy of Sentinel Hub Playground), VAAC ash advisories (http://www.bom.gov.au/), and Global Volcanism Program bulletin reports (Global Volcanism Program, 2017; 2018; 2019).

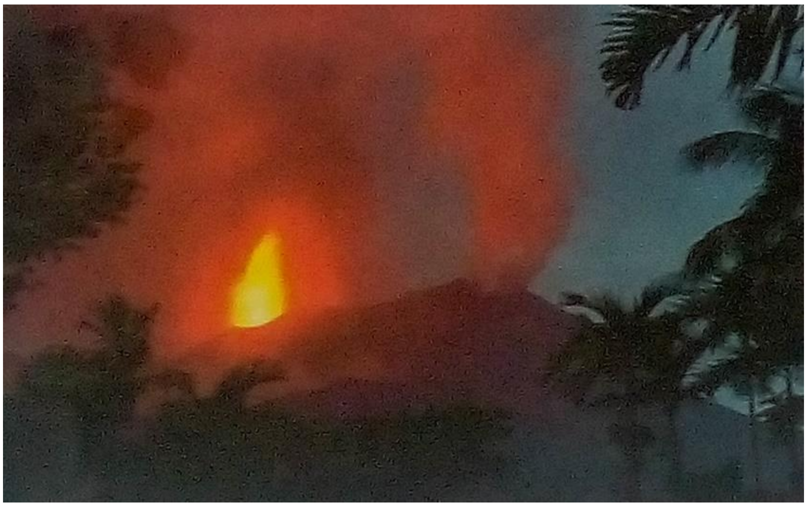

Figure S2: Eruption from Main Crater on 6 November 2019. The sustained lava fountain at Main Crater began less than one month after the reappearance of a thermal anomaly at this crater in Sentinel-2 satellite images. Image credit to Ryan Webb, Manam resident (personal communication).

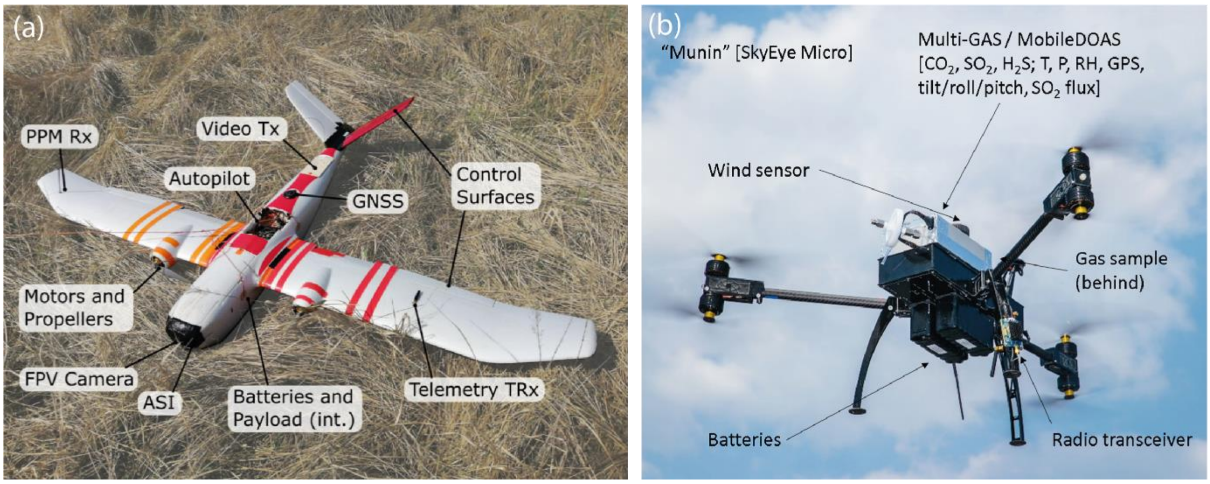

Figure S3: Annotated diagrams of the Unoccupied Aerial Systems (UAS). (a) Fixed-wing 'Titan' aircraft. Image credit: K. Wood; (b) Multi-rotor 'Munin' aircraft in Y6 configuration. Image Credit: M. Wordell. See Material and Methods for more details.

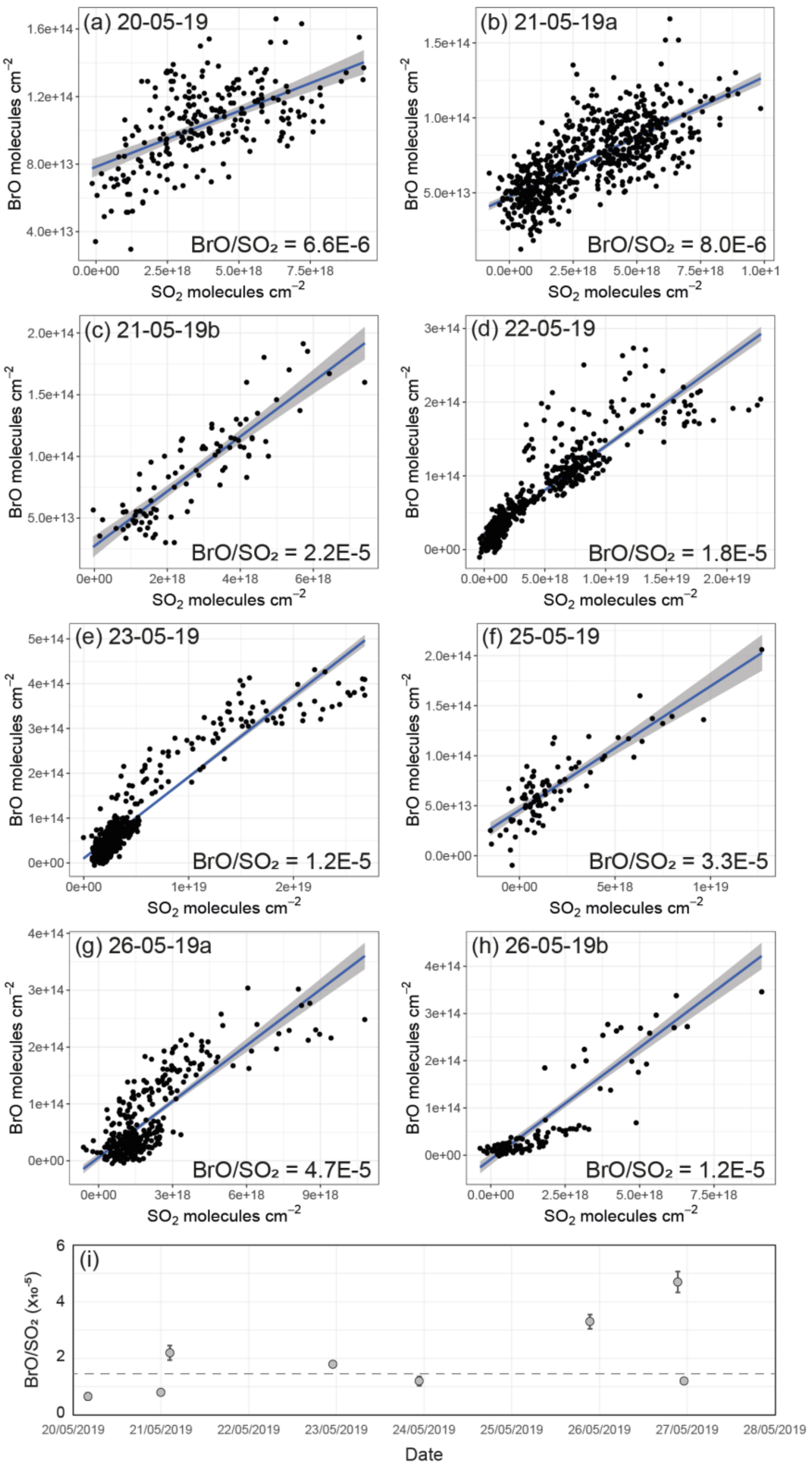

Figure S4: BrO/SO_2 molar ratios. BrO-SO_2 regression scatterplots for (a–h) 20–26 May 2019; all times in UTC. Molar gas ratios are determined by least squares linear regression (solid blue line). Grey shaded region represents the 95% confidence bounds on the regression. (i) Timeseries of molar BrO/SO_2 ratios through the campaign with 95% confidence intervals. Median value is BrO/SO_2 = 1.5 × 10-5 (grey dashed line).

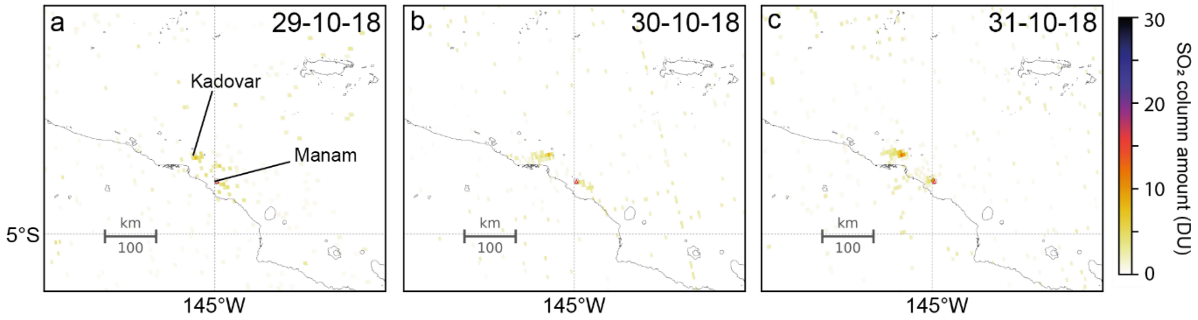

Figure S5: Satellite retrievals of SO_2 mass burden for the period 29–31 October 2018, measured by TROPOMI and interpolated to a plume altitude of 3 km. Two weak SO_2 sources are visible in the retrieved field of view, corresponding to Manam and Kadovar volcanoes (annotated). Colour scale is in Dobson Units (DU), proportional to the number of molecules in a square centimetre of atmosphere.

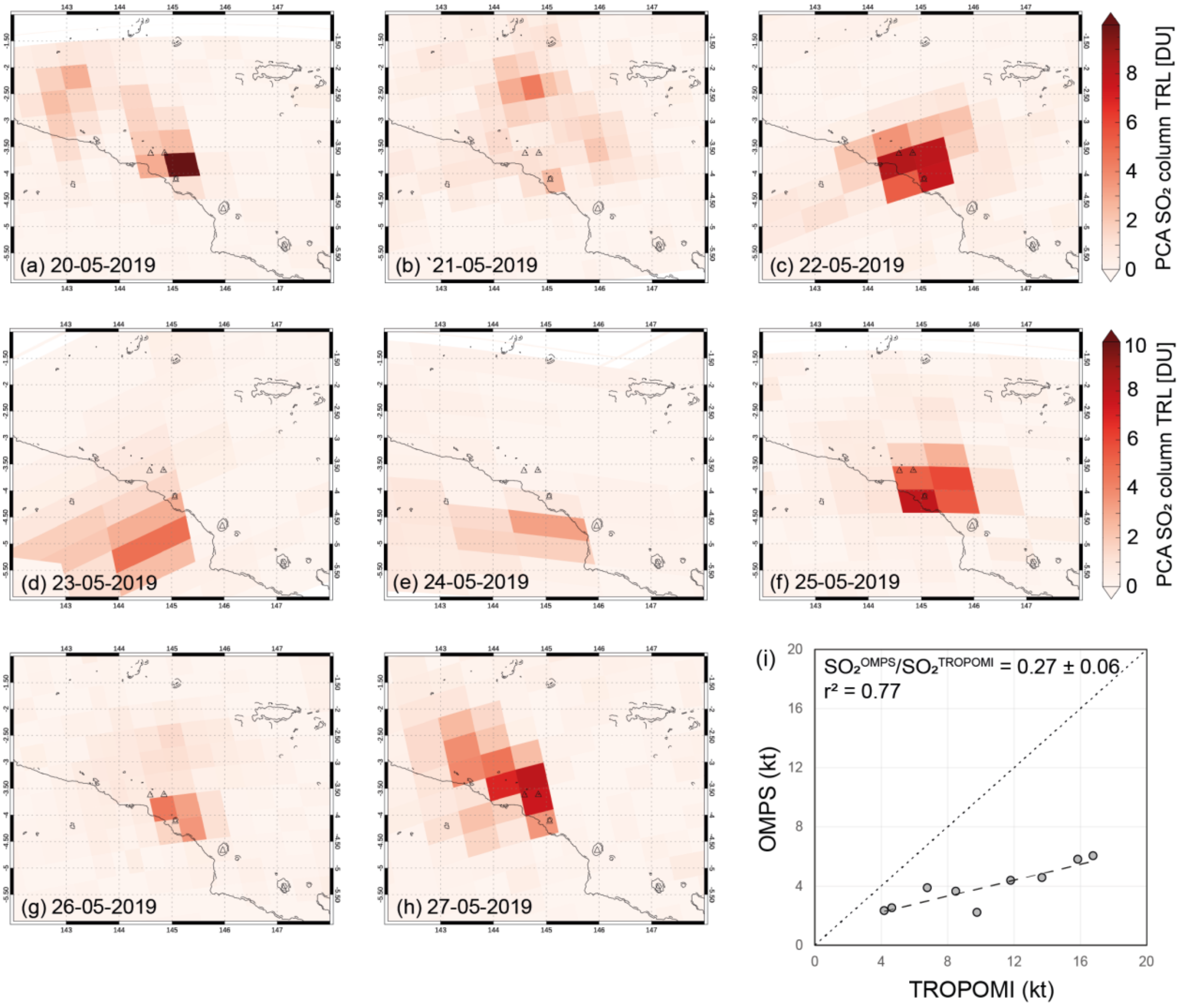

Figure S6: Ozone Mapping and Profiler Suite (OMPS) observations: (a-h) Maps of SO_2 column density for the campaign period 20–27 May 2019, measured by OMPS and interpolated to a plume altitude of 3 km. Colour scale is in Dobson Units (DU). (i) Comparison of OMPS and TROPOMI mass loadings for the period of the field campaign.

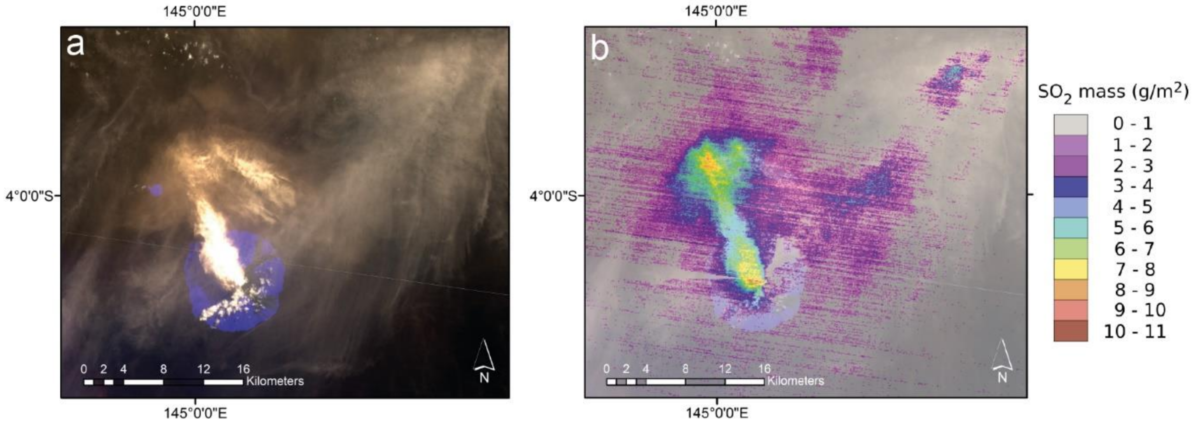

Figure S7: ASTER satellite retrieval; (a) Raw data from overpass of the Advanced Spaceborne Thermal Emission and Reflection Radiometer (ASTER) satellite sensor on 22 May 2019; (b) Map of SO_2 column densities from IR retrieval.

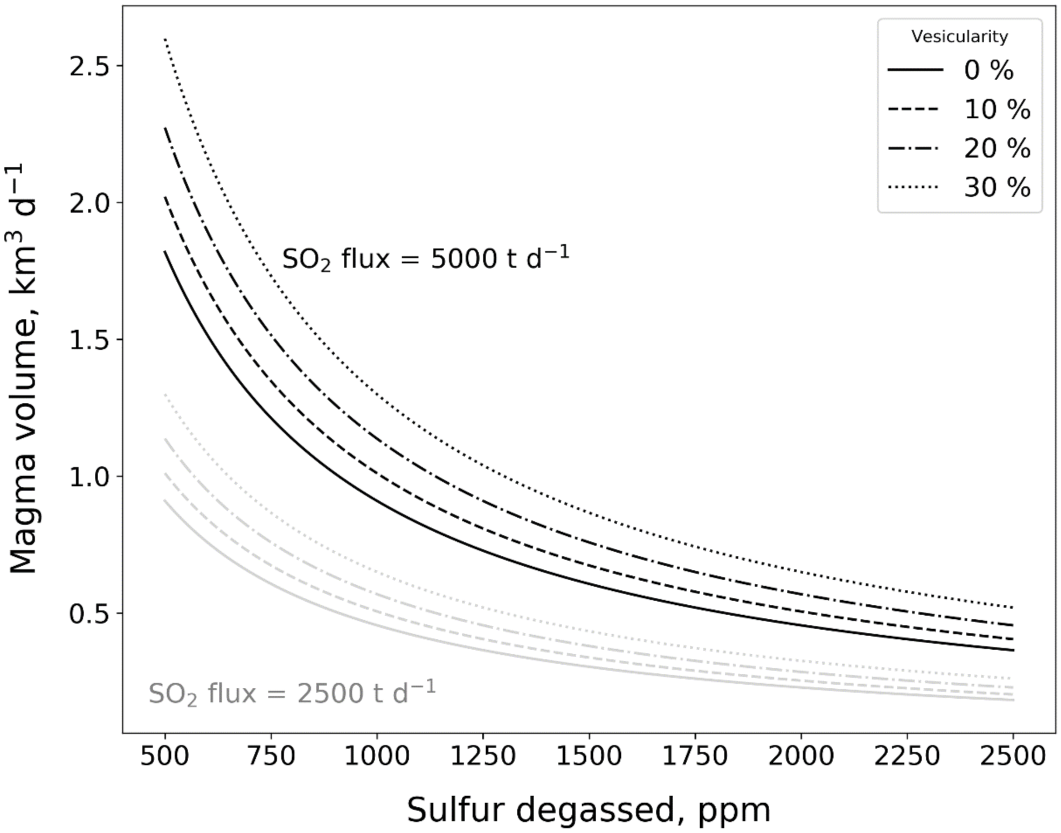

Figure S8: Modelled magma volumes required to produce observed SO_2 fluxes. See Appendix A for details of method, input parameters and assumptions.

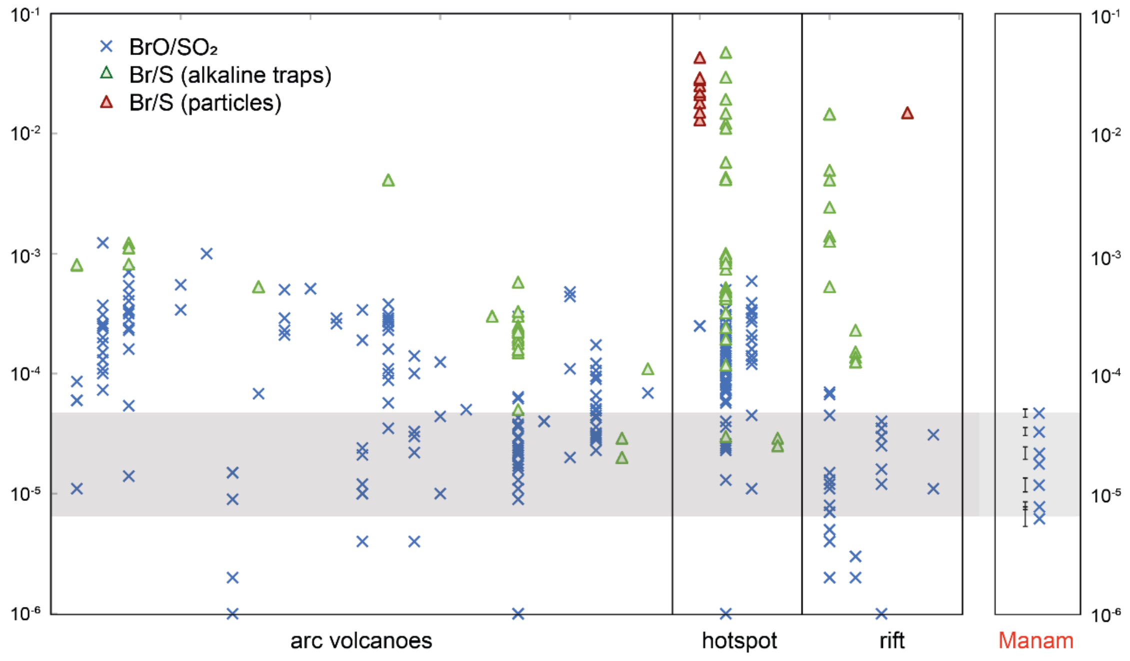

Figure S9: BrO/SO$_2$ ratios in a global context. Comparison of BrO/SO$_2$ ratios measured at Manam (this study) to BrO/SO$_2$ and Br/S ratios measured at volcanoes in different tectonic settings (data from Gutmann et al.[72]). Shaded region highlights the range of measurements for Manam, to aid comparison. Error bars (95% confidence intervals) for each Manam datapoint are shown above the symbol for clarity.

Calculation of magma volumes required to generate the observed SO_2 flux

We determine the volume of magma (in km^3) required to produce (a) 5000 t d^{-1} and (b) 2500 t d^{-1} SO_2 according to equation A1. We vary the input parameters over a reasonable range of values to explore the dependence of the final result on each, due to the lack of petrological constraints on the magma properties at Manam. In this calculation, we assume a magma density of 2750 kg m^{-3} and vary magma vesicularity from 0 to 30 %. We also vary the amount of sulfur degassed from 500 to 2500 ppm (equivalent to 0.05 to 0.2 wt% S). We do not make any explicit correction for crystallinity.

$$V = \frac{f}{(c\,\rho\,\gamma\,\Delta S)} \times 10^{-9} \qquad \text{(equation A1)}$$

V = magma volume (km^3)

f = measured SO_2 flux (converted from t d^{-1} to kg d^{-1})

c = constant for S to SO_2 conversion (c = 2)

ρ = magma density (2750 kg m^{-3})

γ = vesicularity (expressed as melt fraction; i.e. 1 = 0% vesicularity, 0.7 = 30% vesicularity)

ΔS = degassed sulfur (x ppm $\times 10^{-6}$; here varied from x = 500 to x = 2500 ppm)

The results are shown in Figure S8.
